# Altered functional connectivity underpins cognitive changes in chronic spinal cord injury

**DOI:** 10.1093/braincomms/fcag050

**Published:** 2026-02-23

**Authors:** Jothini Sritharan, Lorenzo Diana, Vanessa Vallesi, Nicola Brunello, Lukas Feuring, Jessica Japheth Ugowe, Rahel Oertli, Inge Eriks-Hoogland, Anke Scheel-Sailer, Valentina Moro, Rajeev Kumar Verma, Giuseppe Angelo Zito

**Affiliations:** Swiss Paraplegic Research, Nottwil 6207, Switzerland; Faculty of Health Sciences and Medicine, University of Lucerne, Lucerne 6005, Switzerland; Swiss Paraplegic Research, Nottwil 6207, Switzerland; Swiss Paraplegic Research, Nottwil 6207, Switzerland; Support Centre for Advanced Neuroimaging (SCAN), Institute for Diagnostic and Interventional Neuroradiology, Inselspital, Bern University Hospital, University of Bern, Bern 3010, Switzerland; Swiss Paraplegic Research, Nottwil 6207, Switzerland; ARTORG Center for Biomedical Engineering Research, University of Bern, Bern 3008, Switzerland; Swiss Paraplegic Research, Nottwil 6207, Switzerland; Swiss Paraplegic Research, Nottwil 6207, Switzerland; Faculty of Health Sciences and Medicine, University of Lucerne, Lucerne 6005, Switzerland; Department of Paraplegiology, Swiss Paraplegic Centre, Nottwil 6207, Switzerland; Faculty of Health Sciences and Medicine, University of Lucerne, Lucerne 6005, Switzerland; Department of Paraplegiology, Swiss Paraplegic Centre, Nottwil 6207, Switzerland; Swiss Paraplegic Research, Nottwil 6207, Switzerland; Faculty of Health Sciences and Medicine, University of Lucerne, Lucerne 6005, Switzerland; Center for Rehabilitation & Sports Medicine, Inselspital and Berner Reha Zentrum, Bern University Hospital, University of Bern, Heiligenschwendi 3625, Switzerland; Department of Human Sciences, University of Verona, Verona 37129, Italy; Department of Rehabilitation, IRCSS Sacro Cuore Don Calabria, Negrar 37024, Verona, Italy; Swiss Paraplegic Research, Nottwil 6207, Switzerland; Faculty of Health Sciences and Medicine, University of Lucerne, Lucerne 6005, Switzerland; Department of Radiology, Swiss Paraplegic Centre, Nottwil 6207, Switzerland; Swiss Paraplegic Research, Nottwil 6207, Switzerland; Faculty of Health Sciences and Medicine, University of Lucerne, Lucerne 6005, Switzerland

**Keywords:** spinal cord injury, cognitive impairment, executive functions, functional connectivity, brain reorganization

## Abstract

Individuals with spinal cord injury are subject to a higher risk of cognitive impairment, with executive functions being amongst the most affected ones. Even though the occurrence of cognitive impairment following spinal cord injury has been confirmed in several studies, the related alterations in the brain are not known yet. This prospective observational study aims to tackle this issue by investigating the neural correlates of executive functions in spinal cord injury. Twenty-six individuals with chronic spinal cord injury (mean age = 45.8 ± 11.0 years, mean time since injury = 13.3 ± 11.1 years) and 26 age- and sex-matched non-injured controls (mean age = 43.1 ± 11.5 years) performed a phonemic verbal fluency task as a proxy for executive functions during functional magnetic resonance imaging. We compared task performance, as well as differences in brain activity and seed-based functional connectivity between groups using general linear models. Additionally, we correlated performance and functional connectivity using Pearson correlation. Performance on the verbal fluency task was significantly lower (15% less correct words) in individuals with spinal cord injury, compared to non-injured controls (*P* = 0.02). For the imaging analysis, two individuals with spinal cord injury were removed due to excessive motion and non-attentiveness during the imaging task, leading to a sample size of 24 for the spinal cord injury group. There was significantly lower activity in the right putamen in individuals with spinal cord injury compared to non-injured controls (*P* = 0.015 corrected with family-wise error). Functional connectivity between the left insula and the superior frontal gyrus was lower in spinal cord injury individuals compared to controls (*P* < 0.001 corrected with family-wise error). In spinal cord injury individuals, verbal fluency performance negatively correlated with functional connectivity between the right insula and the right postcentral gyrus (*r* = −0.82, *P* = 0.001 corrected with family-wise error), and between the left putamen and the bilateral precentral gyrus (*r* = −0.74, *P* = 0.002 corrected with family-wise error). Altered brain activity and functional connectivity were found in regions typically associated with executive functions in individuals with spinal cord injury. As the insula acts as a switch between brain networks, including the central executive network, to reallocate cognitive resources, its altered functional connectivity with prefrontal areas may be linked to deficits in executive functions in spinal cord injury. Interestingly, the functional connectivity of sensorimotor regions correlated with verbal fluency performance, suggesting an interference of the disrupted sensorimotor domain with cognitive functions.

## Teaser

Sritharan *et al.* find altered verbal fluency, brain activity and functional connectivity in regions typically associated with executive functions in individuals with spinal cord injury. The widespread brain changes may be linked to deficits in executive functions in spinal cord injury.

## Graphical Abstract

**Graphical Abstract:**
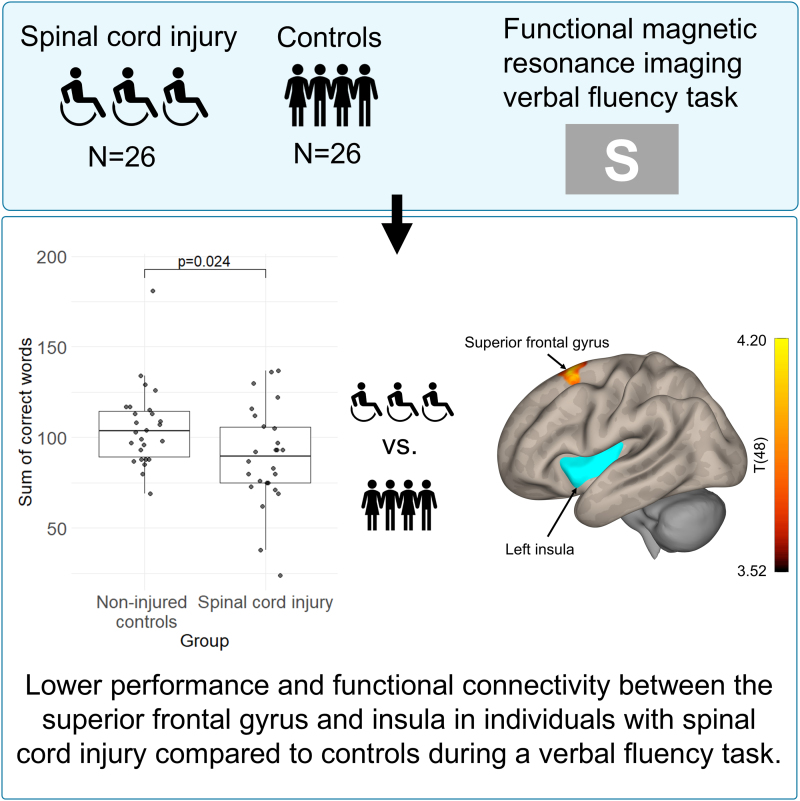

## Introduction

Spinal cord injury (SCI) is a devastating event that can cause long-lasting impairment.^1^ Consequences of a SCI vary from altered organ function (such as bladder and bowel function), development of secondary health conditions (such as urinary tract infection, pressure injury and depression), to activity limitations (e.g. reduced mobility) and interferences with participation in daily life.^1-6^ Changes in cognitive functions are often reported by clinicians after SCI, playing a relevant role not only in the rehabilitation process, but also in the long term participation.^7^

In recent times, SCI-induced cognitive impairment has received considerable attention. People with SCI have a 13-fold higher risk of suffering from cognitive impairment than matched non-injured individuals.^8^ Traumatic brain injury (TBI) is diagnosed in around 60% of SCI cases, and could partially explain the cognitive deficits.^3,9^ Interestingly, cognitive impairment in SCI has been observed even in the absence of TBI,^10-13^ with a prevalence estimated between 10% and 40%.^14^ Other contributing factors to cognitive impairment in SCI are autonomic dysfunctions, psychiatric disorders, substance abuse, medication side effects, pain, neuroinflammation and sleep apnoea.^3,15,16^ If cognitive impairment remains undetected, the resulting behaviour may be interpreted as lack of motivation or non-compliance in rehabilitation settings.^17,18^ Moreover, cognitive impairment is detrimental to self-care and successful reintegration into society,^18-20^ and may lead to a feeling of helplessness and distress when transitioning back to the community.^21,22^

Amongst the cognitive domains impaired in SCI are memory, processing speed, attention and executive functions,^3,23^ the latter being evidenced as the most affected ones in a recent systematic review.^24^ Executive functions play a crucial role in everyday life, and are involved in planning, decision-making, developing strategies, suppressing habits, shifting attention between tasks, reasoning and problem-solving.^25-27^ Verbal fluency tasks are amongst the most common tests to assess executive functions, and measure a person’s ability to retrieve words that meet certain criteria.^28,29^ Phonemic verbal fluency, for instance, requires the participant to retrieve words starting with a specific letter within a given time window.^28,30^ It requires the inhibition of the natural retrieval of words based on associations, which poses a high demand for executive control.^30,31^ Several studies have found that verbal fluency is impaired in SCI,^10,11,32^ but these studies did not investigate the underlying changes in the brain. In fact, disrupted sensorimotor pathways after SCI trigger a functional reorganization in the brain.^33,34^ Whilst alterations affecting the primary somatosensory and motor cortex have been widely investigated in previous studies,^35-38^ functional changes in networks involved in cognition in SCI, such as the attentional networks, have also been observed.^39^ Although we have gained more insights into cognitive function after SCI, the brain changes linked to cognitive processes following SCI still remain unclear.

Using non-invasive imaging techniques, such as functional magnetic resonance imaging (fMRI), neural correlates of cognitive impairment can be studied. In fMRI, the blood-oxygen-level-dependent (BOLD) signal is measured, which is sensitive to the regional deoxygenated haemoglobin concentration, and linked to neuronal activity.^40^ With task-based fMRI, brain activity and the temporal correlation of brain signals,^41^ i.e. functional connectivity (FC), can be assessed in different brain regions during the execution of cognitive tasks. To date, there are only few studies examining changes in the brain during cognitive tasks in SCI.^12,13,42,43^ In the study by Lucci *et al*., for instance, response inhibition was assessed in individuals with SCI and controls, while recording their brain activity with electroencephalography (EEG), and altered event-related potential in the prefrontal cortex was found in SCI.^43^ In further EEG studies, impaired cognitive functions, including body representation and inhibitory functioning, were shown in individuals with SCI, together with altered event-related potentials.^12,42^ These studies have mainly focused on alterations in event-related potentials or local cerebral blood flow in individuals with SCI, but did not discuss changes in specific brain regions,^13^ nor did they investigate the FC between different regions. Indeed, this information is crucial for the understanding of cognitive processes, which often involve the interaction of several brain regions.^44^ Investigating the neural correlates of cognitive tasks could aid in advancing our understanding of the pathophysiological changes associated with cognitive impairment after the injury, and inform targeted interventions to improve cognition.

The goal of the present study was to advance our understanding of cognitive changes after SCI by investigating the neural correlates of cognitive impairment with respect to executive functions. To this end, we conducted a task-based verbal fluency fMRI study and identified patterns of FC linked to verbal fluency in individuals with and without SCI. Our hypothesis was that functional reorganization following SCI extends beyond the sensorimotor cortex and involves neural networks associated with cognitive functions, such as verbal fluency.

## Materials and methods

### Study design and ethical approval

This prospective observational study follows a case-control design. The reporting of this study was based on the Strengthening the Reporting of Observational Studies in Epidemiology (STROBE) guidelines. The study was approved by the local ethics committee of Northwest and Central Switzerland (EKNZ, ID: 2023-02071). All participants provided written informed consent before participating in the study in accordance with the Declaration of Helsinki and guidelines of good clinical practice. The study was preregistered on ClinicalTrials.gov (ID: NCT06309888).

### Study participants

The recruitment period lasted from February 2024 to November 2024. SCI participants were recruited from the Swiss Paraplegic Centre in Nottwil, Switzerland. Participants without SCI were recruited through advertisements on the internal intranet of the Swiss Paraplegic Group, social media, local newspaper and word of mouth.

Study participants included 26 persons with SCI and 26 age- and sex-matched non-injured controls. With respect to the age- and sex-matching, for each enrolled SCI participant, a non-injured control of the same sex who was up to 5 years younger or older than that SCI individual was selected. The sample size was determined *a priori* based on the effect size of a previous study investigating verbal fluency in SCI using the software G*Power (version 3.1) (see Supplementary material for detailed power analysis).^32,45^ The inclusion criteria for both groups were: (i) age between 18 and 60; (ii) German native speaker, and additionally for the SCI group (iii) a diagnosis of traumatic SCI; (iv) time since injury longer than 1 year and (v) American Spinal Injury Association Impairment Scale (AIS) grade A, B, C and D. Exclusion criteria for both groups were: (i) contraindication for MR examinations (e.g. cardiac pacemaker etc.); (ii) pregnancy; (iii) diagnosis of other psychiatric or neurological disorders; (iv) alcohol and/or drug abuse; (v) insufficient hand function and (vi) diagnosis of TBI based on a radiological assessment and, for cases with a high-intensity trauma (e.g. car accident), on a standardized neuropsychological assessment. In particular, we excluded individuals with visible intracranial haemorrhages, midline shifts and/or hydrocephalus on anatomical images (e.g. computed tomography of the head and susceptibility-weighted MRI), as confirmed by an experienced radiologist (RKV). We only included individuals with traumatic SCI to keep the study sample as homogeneous as possible and to have a clear onset date of the injury.^46-49^

### Verbal fluency task

**Figure 1:**
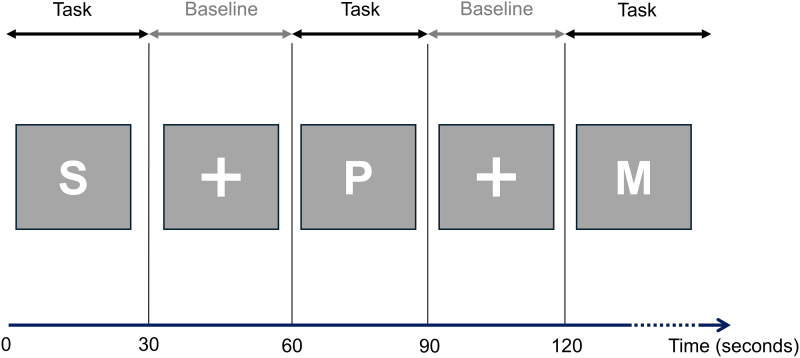
**Design of the phonemic verbal fluency task in the magnetic resonance imaging scanner.** During the task block, a letter was displayed on a screen and participants were instructed to silently produce words starting with the letter for 30 s. During the baseline condition, a fixation cross was shown on the screen and participants had to silently repeat the word ‘Kreuz’ (‘cross’ in German) for 30 s.

Participants underwent an fMRI measurement including a phonemic verbal fluency task, based on the phonemic Regensburger Wortflüssigkeitstest (RWT).^50^ The paradigm was developed in accordance with previous verbal fluency fMRI studies.^51-53^ The task was presented on a screen in a block design and consisted of an alternation of task and baseline. During the task block, a letter was shown on the screen, and participants silently produced as many words as possible, starting with the given letter, within 30 s (see Fig. 1). During the baseline block, a cross was displayed for 30 s, and participants had to silently repeat the word ‘Kreuz’ (‘cross’ in German). This procedure was conducted for seven letters specified in the RWT,^50^ i.e. ‘B’,‘G’,‘K’,‘M’,‘P’,‘R’ and ‘S’, and the order of the letters was randomized for each participant using https://randomizer.org/. To ensure that participants were alert during the task, after completing four and seven letters the participants were asked to press a button on a response device within 10 s. The task was implemented using PsychoPy® (https://www.psychopy.org/, version 2023.2.3). After an hour, the verbal fluency task was repeated overtly outside the scanner to assess performance, following a similar study design, as described by Nair *et al*.^51^ Thereby, the task duration per letter was 1 min and the letters were the same as those used inside the scanner. The sum of correct words across the seven letters and the number of below-average performances based on the normative data of the RWT, which were corrected for age and education, were used as outcome measures for verbal fluency performance. To ensure adequate test-retest reliability, we conducted a pilot measurement before the start of the study, and verified that the intraclass correlation coefficients between two sessions of the verbal fluency task at a time interval of 1 hour were sufficiently high (Supplementary material and Supplementary Fig. 1). We also investigated potential memory effects by determining the percentage of words that were repeated during the second testing.

### Clinical assessment and questionnaires

A series of questionnaires was administered, including questions on general demographic information and medication intake, State-Trait Anxiety Inventory (STAI)^54^ and Numeric Pain Rating Scale (NPRS)^55^ questionnaires. For participants with SCI, the International Standards for Neurological Classification of Spinal Cord Injury^56^ (ISNCSCI) examination was conducted by a certified examiner.

### Image acquisition and processing

The MRI data were acquired in a 3T Philips Achieva scanner with a 32-channel head coil. The acquisition protocol consisted of (i) a structural 3D T1-weighted scan with echo time (TE) = 3.7 ms, repetition time (TR) = 8.1 ms, flip angle = 8° and field of view of 256 × 256 × 180 mm^3^ and (ii) a functional scan with TE = 29 ms, TR = 2.9 s, flip angle = 90°, voxel size = 3 × 3 × 3 mm^3^, field of view of 240 × 240 × 159 mm^3^ and 152 repetitions. The measurement duration for the two sequences was around 10 min.

Images were preprocessed and analysed with CONN release 22.a^57^ and Statistical Parametric Mapping^58^ (SPM12) (http://www.fil.ion.ucl.ac.uk/spm) running under MATLAB R2020a (The MathWorks Inc., Natick, Massachusetts). The functional images were realigned with the correction of susceptibility distortion interactions. Slice timing correction, outlier detection of scans with a framewise displacement above 0.9 mm or global BOLD signal changes above 5 standard deviations, normalization to Montreal Neurological Institute (MNI) space, segmentation, and smoothing with a Gaussian kernel of 8 mm full-width half maximum were applied.

Functional data were denoised, including the regression of potential confounding effects characterized by white matter and cerebrospinal fluid time series, motion parameters and their first-order derivatives, outlier scans, session and task effects, linear trends within each session and a high-pass frequency filtering of the BOLD signal above 0.008 Hz. We further included the task and baseline conditions as potential confounders in the denoising step, and reduced the influence of constant task-related co-activation in the BOLD signal.^59^ Data quality metrics are displayed in the Supplementary material and were comparable between groups (Supplementary Figs 2 and 3). Outliers with excessive movement during the scanning session were individuals for whom the mean motion deviated by more than three interquartile ranges above the third or below the first quartile. Participants who exceeded this threshold were excluded.

### Imaging and functional connectivity analysis

The imaging analyses consisted of two parts: investigating group differences in brain activity and seed-based FC, respectively. For the analysis on brain activity, a general linear model (GLM) was implemented in SPM, testing for differential BOLD signal in the task condition between individuals with SCI and individuals without SCI. *P*-values were family-wise error corrected (p_FWE_). We also computed activation maps for each group individually and verified that regions typical for phonemic verbal fluency^60^ were activated in both groups (Supplementary Figs 4 and 5, Supplementary Tables 1 and 2). For the seed-based FC analysis, 11 seeds (i.e. bilateral inferior frontal gyrus, bilateral insula, bilateral anterior cingulate gyrus (ACG), left thalamus, right cerebellum crus I, left precuneus, right caudate and left putamen) from a previous meta-analysis were used, which identified regions being active during phonemic verbal fluency tasks in healthy individuals.^60^ BOLD signal time series were extracted from these regions using the Harvard-Oxford atlas.^61^ In the first-level analysis, FC values were calculated as Fisher-transformed bivariate correlation coefficients from a weighted GLM, and used to determine condition-specific FC between the time series of each seed and every voxel in the brain during the task condition.^59^ Individual scans were weighted by a boxcar function characterizing each condition convolved with an SPM canonical haemodynamic response function and rectified. For the second-level analysis, a GLM was implemented for each single voxel, using the group variable (SCI versus non-injured controls) as the independent variable and the FC of that voxel per subject as the dependent variable. Age was used as a covariate in the analysis. Voxel-level hypotheses were assessed using multivariate parametric statistics with random-effects across subjects and sample covariance estimation across multiple measurements. Inferences were carried out at the level of individual clusters. Reported FC values represent the mean of FC values of the whole cluster.^59^ Cluster-level inferences were estimated based on the Gaussian Random Field theory,^62,63^ and results were corrected for multiple comparisons using FWE correction *P*_FWE_ < 0.05 at a cluster-level. As 11 different seeds were used, an additional Bonferroni correction was applied to the FWE corrected *P*-values, leading to a new significance threshold at *P*_FWE_BF_ < 0.0045.

### Statistical analysis

Statistical analyses of clinical and behavioral data were performed in R (version 4.4.1). The following analyses were performed: (i) a comparison of demographic and clinical characteristics between the groups, (ii) a comparison of verbal fluency performance between groups, (iii) a comparison of the number of below-average performances between the groups and (iv) a correlation of verbal fluency performance with FC values.

#### Group comparisons of behavioral and clinical data

Differences in categorical clinical and demographic data between SCI individuals and non-injured controls were compared using Chi-square tests, and differences in continuous data were determined with a Wilcoxon rank sum test. Group differences in verbal fluency performance between SCI and non-injured controls were assessed using Wilcoxon rank sum tests due to the violation of normality and homogeneity of variance assumptions. Model assumptions were tested with the Shapiro–Wilk test and Levene's test, respectively. The test statistic of the Wilcoxon rank sum test is denoted by *W* and of the Chi-square test by $\chi^{2}$.

To assess the clinical relevance of verbal fluency differences, and to account for the potential effect of age and education level on cognitive performance, we compared each individual’s word number with the normative data of the RWT,^50^ which was adjusted for education level and age. Normative data are available for the five letters S, P, M, K and B.^50^ In this way, each subject and each letter were assigned a percentile rank, describing the percentage of individuals from the norm population who performed equally or worse for that specific letter in the same age and education group.^50^ If a percentile rank was smaller than 10, then the performance of the subject for this letter was classified as below-average performance, according to Lezak.^64^ With this, a new outcome variable was assigned to each subject, denoting the number of below-average performances, ranging from 0 to 5. The number of below-average performances was compared between individuals with and without SCI using a Wilcoxon rank sum test.

Subgroup and regression analyses addressed: (i) differences in the ISNCSCI between body sides (right versus left) and (ii) group differences in verbal fluency performance, based on AIS levels (AIS A versus AIS D), medication intake (pain medication and antimuscarinics) and lesion levels (cervical versus thoracic) using the same methods as described in this section. Potential effects of clinical variables on cognitive performance were examined.

#### Correlation between verbal fluency performance and functional connectivity

Lastly, correlations between verbal fluency performance and FC were determined in individuals with and without SCI separately using Pearson correlation. Seed-based FC values were correlated with verbal fluency performance using age as a covariate. As for the imaging analysis, Bonferroni correction was applied to the FWE corrected *P*-values to account for the number of seeds, resulting in the new significance threshold at *P*_FWE_BF_ < 0.0045. The correlation coefficient is denoted by *r*.

## Results

### Participant characteristics

Twenty-six individuals with traumatic SCI (mean age = 45.8 ± 11.0 years, mean time since injury = 13.3 ± 11.1 years) and 26 without SCI (mean age = 43.1 ± 11.5 years) participated in the study (Table 1). Eleven individuals with SCI were characterized with an AIS level A, 1 with AIS B, 2 with AIS C and 12 with AIS D. The ASIA motor score was significantly lower (*P* = 0.009) on the left compared to the right body side in individuals with SCI (Supplementary Fig. 6). All participants were included in the behavioural analysis, and for the imaging analysis two SCI subjects had to be removed due to excessive motion and non-attentiveness during the imaging task, leading to a sample size of 24 (mean age = 46 ± 11.3 years, mean time since injury = 13.1 ± 10.8 years) for the SCI group. There was no significant difference in age (*P* = 0.290, *W* = 280), sex distribution (*P* = 1, $\chi^{2}$ = 0), handedness (*P* = 0.193, $\chi^{2}$ = 1.7) and highest degree of education (*P* = 0.125, $\chi^{2}$ = 7.22) between the two groups. Individuals with SCI showed a significantly higher level of pain (*P* < 0.001). The two groups did not differ in anxiety level. There was a significant difference in the medication intake of bladder medication, antimuscarinics and pain medication between the two groups. The specific agents contained in each of the medication group is described in the Supplementary material. Detailed clinical and demographic characteristics for the SCI individuals are described in Supplementary Table 3.

**Table 1:** Clinical and demographic characteristics

	Spinal cord injury (*n* = 26)	Non-injured controls (*n* = 26)	Test statistics	*P*-value
Sex (male/female)	18/8	18/8	$\chi^{2}$ = 0	1
Age [years, mean ± SD]	45.8 ± 11.0	43.1 ± 11.5	*W* = 280	0.290
Handedness (right/left)	21/5	25/1	$\chi^{2}$ = 1.7	0.193
NPRS current pain [mean ± SD]	2.7 ± 2.2	0.3 ± 0.5	*W* = 99	**<0.001**
STAI anxiety state [mean ± SD]	37.5 ± 8	33.73 ± 5.7	*W* = 246	0.090
STAI anxiety trait [mean ± SD]	37.2 ± 8.6	32.9 ± 5.7	*W* = 238	0.070
Level of injury				
C1–C4	4 (15.4%)	NA	NA	-
C5–C8	8 (30.8%)			
T1–S5	14 (53.8%)			
ISNCSCI				
Total motor score [mean ± SD]	72.2 ± 21.1	NA	NA	-
Total light touch score [mean ± SD]	71.5 ± 21.4			
Total pinprick score [mean ± SD]	72.2 ± 23.8			
Medication *n* (%)				
Bladder without antimuscarinics	9 (34.6%)	0 (0%)	$\chi^{2}$ = 8.6	**0.003**
Antimuscarinics	11 (42.3%)	0 (0%)	$\chi^{2}$ = 11.5	**<0.001**
Pain	11 (42.3%)	1 (3.8%)	$\chi^{2}$ = 8.8	**0.003**
High blood pressure	5 (19.2%)	1 (3.8%)	$\chi^{2}$ = 1.7	0.193
Spasticity	5 (19.2%)	0 (0%)	$\chi^{2}$ = 3.5	0.060
Others (thyroid, birth control, acne, rheumatism, HIV, cholesterol, constipation)	7 (26.9%)	3 (11.5%)	$\chi^{2}$ = 7.9	0.700

Note: NA: not applicable.

### Performance in verbal fluency

**Figure 2:**
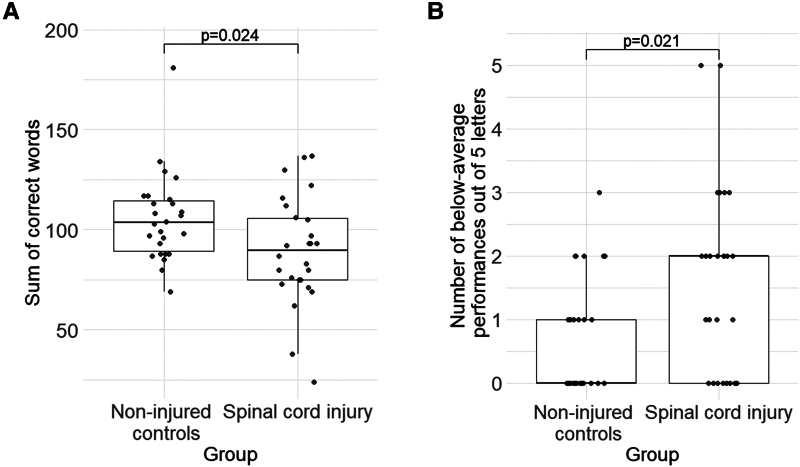
**Boxplots showing differences in verbal fluency performance between individuals with and without SCI.** Single datapoints indicate the performance of individual participants, and the bold horizontal lines in the boxplots indicate the median within each group. Panel A shows a significantly lower sum of correct words using a Wilcoxon rank sum test (*W* = 462, *P* = 0.024) in the SCI group (*N* = 26) compared to the non-injured controls (*N* = 26). The boxplot in panel B shows the number of below-average performances (ranging from 0 to 5) in each of the groups based on the education- and age-adjusted normative data from the RWT. The SCI group was characterized by a significantly higher number of below-average performances compared to the non-injured controls (*W* = 218, *P* = 0.021).

The sum of correct words across the seven letters of the verbal fluency test was significantly lower (*W* = 462, *P* = 0.024) in the group of SCI individuals (89.4 ± 27.2) compared to non-injured controls (105.5 ± 22) (see Fig. 2A). On average, people with SCI produced 15.3% less correct words than the control group. There were significantly more (*W* = 218, *P* = 0.021) below-average performances based on the five letters with normative data in the SCI group (1.6 ± 1.5) compared to the control group (0.7 ± 0.9) (see Fig. 2B). Age- and education-adjusted percentile ranks of the study participants are displayed in Supplementary Fig. 7.

### Imaging results

#### Differences in brain activity and functional connectivity

**Figure 3:**
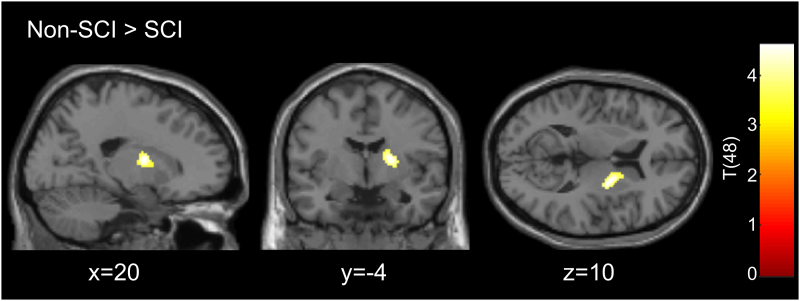
**Group differences in brain activity during the phonemic verbal fluency task using a two-sample *t*-test.** There is a significant cluster (*P* = 0.015, corrected with family-wise error, cluster size = 350 voxels) with a peak in the right putamen, when comparing the blood-oxygen-level-dependent signal between individuals with (*N* = 24) and without SCI (*N* = 26) during a phonemic verbal fluency task. The group differences are displayed on a standard brain. Two SCI subjects were removed from the imaging analysis due to excessive motion and non-attentiveness during the measurement.

There was significantly lower BOLD signal in a cluster containing the putamen, pallidum, caudate and thalamus with peak in the right putamen (*P*_FWE_ = 0.015, cluster size = 350 voxels and peak MNI coordinates = 20, −4 and 10) in the SCI group compared to non-injured controls (see Fig. 3). Further correlation between verbal fluency performance and activity in this cluster in all participants is shown in Supplementary Fig. 8.

**Figure 4:**
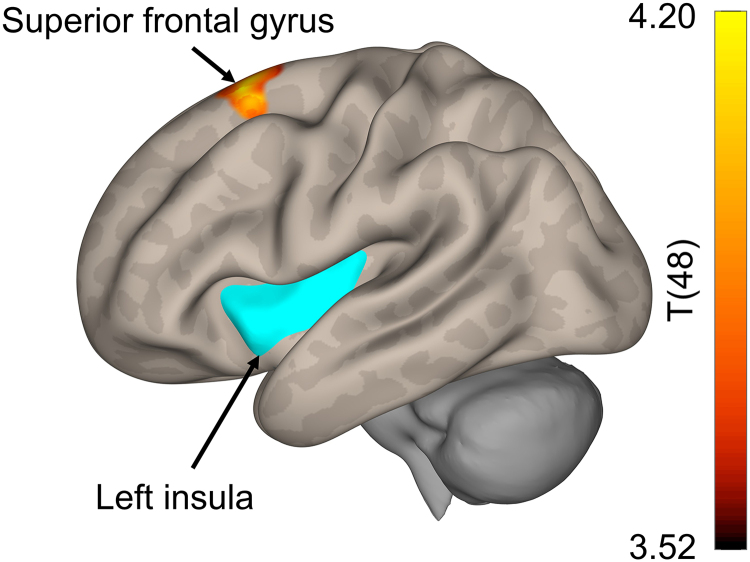
**Group comparison in functional connectivity between individuals with spinal cord injury and non-injured controls using a general linear model.** There was significantly altered functional connectivity (*P* = 0.0002, corrected with family-wise error and Bonferroni, cluster size = 428 voxels) between the left insula (seed) and a cluster containing the left and right superior frontal gyrus between individuals with (*N* = 24) and without spinal cord injury (*N* = 26). Two SCI subjects were removed from the imaging analysis due to excessive motion and non-attentiveness during the measurement.

We also found significantly lower FC between the left insula (seed) and a cluster containing the left and right superior frontal gyrus, in individuals with SCI compared to non-injured controls (*P*_FWE_BF_ = 0.0002, cluster size = 428 voxels and peak MNI coordinates = −18, 16 and 60) (see Fig. 4). None of the other seeds showed significant group FC differences after Bonferroni correction.

#### Correlation between verbal fluency performance and functional connectivity

**Figure 5:**
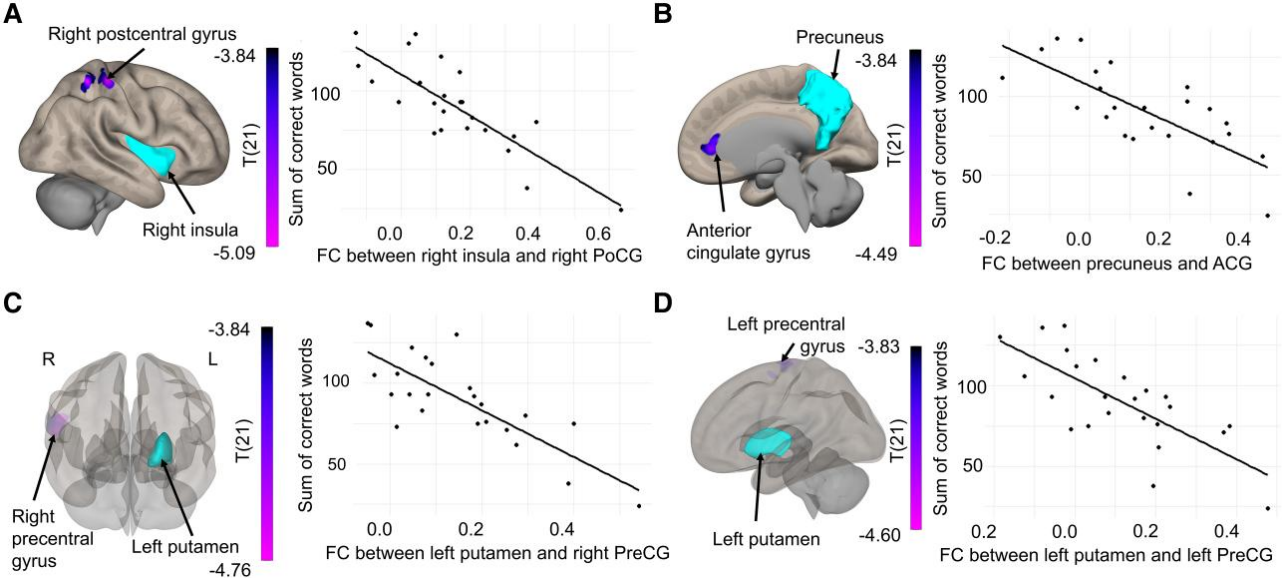
**Location of regions showing significant correlation of functional connectivity (FC) with verbal fluency performance in individuals with spinal cord injury using Pearson correlation.** Panel A shows the correlation between the FC in the right insula and right PoCG with the sum of correct words (*r* = −0.818, *P*_FWE_BF_ = 0.001, corrected with family-wise error and Bonferroni) in individuals with spinal cord injury (*N* = 24). Panel B shows the association between FC in the precuneus and the ACG with verbal fluency performance (*r* = −0.734, *P*_FWE_BF_ = 0.004). The FC between the left putamen and both the right precentral gyrus (PreCG) (Panel C) (*r* = −0.794, *P*_FWE_BF_ = 0.001) and the left PreCG (Panel D) (*r* = −0.736, *P*_FWE_BF_ = 0.002) negatively correlated with the sum of correct words.

In SCI individuals, there was a significant negative correlation between the performance on the verbal fluency task and the FC of the right insula (seed) with the right postcentral gyrus (PoCG) (see Fig. 5A). Similarly, verbal fluency performance negatively correlated with the FC between the precuneus (seed) and the ACG (see Fig. 5B), and with the FC between the left putamen and both the right and the left precentral gyrus (PreCG) (Figs 5C and D). Detailed statistical values for the correlation analysis are displayed in Table 2.

**Table 2:** Significant correlations between seed-based functional connectivity and the sum of correct words in the verbal fluency task in individuals with spinal cord injury

Seed	Functionally connected cluster (percentage of voxels in cluster overlapping with anatomical region)	Peak MNI (*x*, *y*, *z*)	Cluster size (voxels)	*r*-value	*P*_FWE_BF_
Right insula	Cluster 1: right PoCG (68%), right superior parietal lobule (24%)	44, −36, 58	312	−0.818	0.0007
Precuneus	Cluster 2: ACG (84%), right paracingulate gyrus (6%)	−6, 40, 4	231	−0.734	0.0036
Left putamen	Cluster 3: right PreCG (81%), right inferior frontal gyrus (7%)	54, 2, 18	250	−0.794	0.0014
	Cluster 4: left PreCG (49%), left and right supplementary motor area (28%)	−8, −20, 72	242	−0.736	0.0018

Note: *P*_FWE_BF_: *P*-values corrected for family-wise error at the cluster level and additional Bonferroni correction applied to account for the number of seeds, *r*-value: correlation coefficient.

There were no significant correlations between seed-based FC of the 11 seeds with verbal fluency performance in individuals without SCI.

### Effect of contributing factors on cognition

Neither did we find a significant difference in verbal fluency performance, nor in the brain measures for the subgroup analyses AIS A versus AIS D (Supplementary Fig. 9, Supplementary Table 4), cervical versus thoracic SCI (Supplementary Fig. 10, Supplementary Table 5) and SCI individuals taking versus not taking antimuscarinics (Supplementary Fig. 11, Supplementary Table 6). Moreover, when including group, antimuscarinics, pain medication and spasticity medication intake as independent variables in a regression model, there were no significant effects either (Supplementary Table 7). For the subgroup analysis on SCI individuals taking versus not taking pain medication, we did not find differences in verbal fluency performance (Supplementary Fig. 12, Supplementary Table 8), but we found increased FC between the left inferior frontal gyrus (seed) and the precuneus (*P*_FWE_BF_ = 0.0003, cluster size = 335 voxels and peak MNI coordinates = −8, −68 and 52) in the individuals taking pain medication, compared to the SCI individuals taking no pain medication (Supplementary Fig. 13).

## Discussion

In this study, we used task-based fMRI to investigate brain activity and FC differences during a phonemic verbal fluency task, as well as their correlation with verbal fluency performance, in chronic SCI. We demonstrated: (i) lower performance in the verbal fluency task in the SCI group compared to the non-injured controls; (ii) lower BOLD signal during the verbal fluency task in the right putamen in individuals with SCI compared to those without SCI; (iii) lower FC between the left insula and the superior frontal gyrus in the SCI group compared to the non-injured controls and (iv) negative correlation between verbal fluency performance and FC of the following pairs of regions: right insula and right PoCG, precuneus and ACG, and left putamen and bilateral PreCG, in individuals with SCI.

Overall, the SCI group exhibited a higher pain level and higher medication intake in comparison with the group of non-injured controls, which is in line with epidemiological studies.^65-68^ The lower performance in verbal fluency in individuals with SCI lacking a diagnosis of TBI is in accordance with several previous studies.^10,11,32^ The novelty of our study is the investigation of the neural correlates of impaired verbal fluency, an indicator of executive functions, in SCI.

### Between group differences in brain activity and functional connectivity

Individuals with SCI showed lower brain activity in a cluster containing the right basal ganglia and thalamus, with a peak in the putamen, during the verbal fluency task. The basal ganglia are a set of subcortical structures involved in motor initiation and suppression, and are directly connected to the sensorimotor regions.^69,70^ In a voxel-based meta-analysis, grey matter atrophy was shown in the basal ganglia and thalamus in individuals with SCI, indicating that the disruption of sensorimotor pathways may also trigger reorganization in subcortical areas related to the movement domain.^70^ Interestingly, the basal ganglia are not only involved in motor control, but there is also evidence showing their contribution to executive functions.^71,72^ In particular, the involvement of the putamen and the caudate was found in phonemic verbal fluency tasks.^60^ A previous study showed that a smaller grey matter volume in the putamen was associated with lower verbal fluency scores in healthy older adults.^73^ Moreover, the caudate is known to be involved in the suppression of irrelevant words,^74^ a capability highly relevant for the verbal fluency task.

Overall, these findings may suggest that the loss of sensorimotor pathways in SCI may induce changes in connected subcortical motor areas, such as the basal ganglia,^70^ which is observed as lower brain activity during the verbal fluency task. However, due to the cross-sectional design of our study, causality cannot be inferred, and further research is needed to investigate whether cognitive impairment is a consequence of brain changes or a concomitant phenomenon.

Of note, even though verbal fluency and language functions are usually associated with the left hemisphere,^60^ we found differential activity in the right basal ganglia. We exclude the effect of handedness on this right lateralization, as our participants were mostly right-handed. One possible explanation for this lateralization could be the lower ASIA motor score on the left compared to the right side in our SCI sample. As the basal ganglia are not only involved in executive functions, but also in motor control,^69^ the side of the more severe motor impairment after SCI may have an impact on the contralateral brain hemisphere. However, future studies are required to confirm this hypothesis. An alternative explanation for the lateralization of our findings may also be that inhibition is more right lateralized in the brain^75,76^ and our results confirm such a lateralization in SCI as well.

Our study also revealed lower FC between the left insula and a cluster containing the left and right superior frontal gyrus, in individuals with SCI compared to non-injured controls, during the verbal fluency task. The prefrontal cortex is widely known to be involved in executive functions.^72,77,78^ In the context of verbal fluency, the left anterior insula seems to be involved in speech production, articulation and phonation.^60,79,80^ The anterior portion of the insula has been shown to be functionally connected to the prefrontal cortex, and may be involved in higher-order cognitive processes.^81-83^ An integration of interoceptive with cognitive signals occurs in the anterior insula, assigning it the role of a ‘hub’ in the regulation of cognitive processes.^84^ In particular, the insula detects salient stimuli, and switches between other brain networks, such as the central executive network,^82,85-87^ to enable access to cognitive resources.^88^ In the case of SCI, grey matter volume loss has been found in the insula^89,90^ and altered FC was found between the insula and the frontal pole in individuals with SCI compared to non-injured controls.^91^ Based on these findings, there seems to be a disrupted connectivity between the insula and the prefrontal cortex in SCI,^91^ which may affect the gatekeeping function of the insula in the activation of the central executive network, and therefore may impact cognitive processes critical to verbal fluency. Interestingly, we did not find group differences in the insula when comparing the BOLD signal between SCI and non-SCI individuals. As BOLD signal and FC capture two different neurophysiological processes,^41,92,93^ our results may imply that the insula stays engaged on a regional level during verbal fluency, but shows abnormal communication with other executive regions. This may ultimately indicate a disruption on a network level, rather than the mere activity.

### Correlation between verbal fluency performance and functional connectivity

In individuals with SCI, we found that lower verbal fluency performance was associated with higher FC of the precuneus and ACG, which are known to be involved in verbal fluency.^60^ Both the precuneus and the ACG are contributing to high-order cognitive processes and may play a role in processing phonological information.^60,94-96^ A previous study found altered FC with the precuneus and the cingulate gyrus in SCI, which are part of the central executive network.^97^ Thus, our findings further strengthen the link between executive functions and FC in this brain network, confirming the specificity of our imaging results in relation to verbal fluency.

Interestingly, verbal fluency performance in SCI also correlated with FC in regions of the sensorimotor cortex: the right PoCG with the right insula and the bilateral PreCG with the left putamen. Several studies have pointed out brain alterations in the PreCG and PoCG after SCI, which indicate phenomena of brain reorganization, possibly attributed to compensatory mechanisms for the loss of sensorimotor function.^98-101^ A previous resting-state fMRI study found altered FC between the insula and PoCG in individuals with SCI.^91^ Others have further shown increased FC of motor-related regions in SCI, which may reflect over-recruitment of additional brain resources to compensate for the motor deficits.^102,103^ A different fMRI study in SCI found decreased brain activity in cognitive-related regions during a visuomotor imagery task and lower cognitive performance in SCI individuals, suggesting an interaction between the cognitive and the sensorimotor domain.^100^ As the FC between regions of the sensorimotor cortex (PreCG and PoCG) and regions associated with verbal fluency (insula and putamen)^60^ negatively correlated with verbal fluency performance, our results may indicate that disrupted sensorimotor functions in SCI may interfere with cognitive functions, possibly due to phenomena of cortical reorganization, thus resulting in decreased cognitive performance. Future research may confirm this explanation by performing a dual motor-cognitive task in SCI during fMRI. The correlational findings also imply that higher FC between specific pairs of regions is associated with worse cognitive performance in individuals with SCI. These regions only partially overlap with the group differences in FC, and they are part of different resting-state networks,^104-106^ in particular the ventral (right insula) and dorsal attention network (right superior parietal lobule) extending to the somatomotor network (PoCG), and the ventral attention (left putamen) and the somatomotor network (bilateral precentral gyrus). In a previous study, a similar association between increased between-network connectivity and decreased cognitive function was shown in individuals after sleep deprivation.^107^ Therefore, the higher FC in the correlation analysis may indicate that abnormal between-network connectivity in individuals with SCI may reflect maladaptive functional changes, which may be detrimental to cognitive functions.^107-110^

### Effect of contributing factors on cognition

We conducted several subgroup analyses with respect to clinical variables within the SCI group, and investigated the effect of potential contributing factors that may influence cognitive performance. For the majority of clinical variables, we could not find differences in verbal fluency performance and the brain measures, which could also be due to the small sample sizes within each of the subgroups. For instance, we did not find differences between cervical and thoracic SCI, neither in the behavioral nor in the imaging data. This is in line with a previous study, in which no difference in verbal fluency performance was found between tetraplegia and paraplegia.^11^ The lack of differences may also be linked to the fact that in the current study, we only focused on one subdomain of cognition, and a detailed neuropsychological assessment may be required to capture changes across several cognitive domains.^11^ Interestingly, we found differences in FC during the verbal fluency task between the left inferior frontal gyrus and the precuneus between SCI individuals taking pain medication, compared to SCI individuals not taking pain medication. These two SCI groups, however, did not differ in their pain level. In a recent meta-analysis, it was shown that chronic pain (irrespective of SCI) generally engages the insula, the superior frontal gyrus and the superior temporal gyrus.^111^ The prefrontal cortex plays a crucial role in pain processing, especially for the affective and cognitive dimensions of pain.^112-114^ A previous verbal fluency study found altered brain activity in the prefrontal cortex in individuals with chronic pain compared to healthy controls.^115^ We observed a partial overlap between brain regions engaged in chronic pain (insula and superior frontal gyrus) and the regions in which we found differences during the verbal fluency task in individuals with SCI compared to non-injured controls. It may be possible that these patterns of activation reflect the presence of pain. However, the differences in the BOLD signal in the basal ganglia and the correlational findings with motor-related regions seem to be more specific for SCI itself and not pain. Thus, it cannot be ruled out that the lower cognitive performance observed in the SCI group may be the consequence of different factors, including medication side effects, pain and brain reorganization. Future studies could investigate the effect of distinct contributing factors to cognitive impairment, which was outside the scope of the current work.

### Limitations

One limitation of the study is that the verbal fluency task was silently performed inside the scanner to avoid motion artefacts during the measurement, and task compliance could not be guaranteed. However, we implemented an unannounced request to press a button during the measurement to identify subjects who were not attentive during the task. As all included participants, except for one, correctly reacted to this check, it can be assumed that the participants were compliant with the given instructions. To assess performance, the verbal fluency task was repeated one hour after the measurement outside of the scanner, which may introduce a bias due to carryover effects. To address this issue, we conducted a pilot study with healthy volunteers to confirm that repeating the test after 1 h led to an adequate test–retest reliability. However, it cannot be completely ruled out that memory effects may have also contributed to the observed performances. Moreover, the variety of medication taken by individuals with SCI and therefore the potential side effects on cognition^3^ differ significantly, which increases the heterogeneity within the SCI group. Additionally, we did not collect information on the presence of neuropathic pain, and therefore a potential relationship between neuropathic pain and cognitive deficits could not be investigated.^116^ Another limitation of the study is the absence of a control task, targeting a different cognitive domain, such as attention, which would allow us to disentangle changes in brain patterns specific to executive functions from those generally linked to cognitive impairment after SCI. However, verbal fluency performance correlated with brain activity in the putamen across the whole sample (Supplementary Fig. 8), which speaks in favour of the specificity of the task, as the putamen is known to be involved in phonemic verbal fluency.^60^ With respect to the exclusion of participants with TBI with visible damage in the brain, we cannot rule out that someone with an undiagnosed TBI without visible damage in the brain may have participated in the study.

### Therapeutic considerations

This study adds further evidence to the presence of impairment in executive functions in individuals with chronic SCI and points to the related changes in the brain. A study comparing individuals with subacute and chronic SCI found that cognitive functions worsen over time after SCI.^20^ Therefore, it is important that more awareness is raised amongst healthcare professionals that cognitive impairment may develop over time in individuals with SCI,^18^ which is relevant for the interactions with them throughout the rehabilitation process. We suggest that cognitive rehabilitation should be integrated into the rehabilitation program for SCI. Non-invasive brain stimulation, for instance, poses one potential therapy for improving cognitive functioning.^117^ Identifying the neural correlates of cognitive impairment in SCI might be informative for the selection of target brain areas for non-invasive brain stimulation. Techniques such as transcranial direct current stimulation and transcranial magnetic stimulation have been previously shown to improve cognition across different disorders with significant effects.^117^ As we found altered FC with the superior frontal gyrus in the current study, we may speculate that stimulation over this area in SCI may promote cognition. Future studies could investigate the effect of applying non-invasive brain stimulation to the superior frontal gyrus on cognition in SCI. Moreover, pain is a potential contributing factor to cognitive impairment in individuals with SCI,^3^ and a previous longitudinal study in individuals with chronic pain showed that the reduction of pain was linked to a normalization of brain activity during a cognitive task, implying that these brain changes may be reversible.^118^ Therefore, managing pain in individuals with SCI may also help in improving cognitive functioning, and should be further investigated in a longitudinal study.

## Conclusion

In this verbal fluency study, brain activity and FC were found to be altered in brain regions typically associated with executive functions in individuals with chronic SCI compared to non-injured controls. As the insula is considered a hub for controlling executive processes, its altered FC with the superior frontal gyrus may be linked to deficits in executive functions in SCI. Moreover, the FC of the sensorimotor cortex negatively correlated with verbal fluency performance, suggesting that the affected sensorimotor network in SCI may directly interfere with cognitive functions, possibly due to phenomena of cortical reorganization. This study adds evidence that cognitive impairment in SCI is linked to brain reorganization beyond the sensorimotor domain and may serve as a starting point to develop future rehabilitation strategies targeted at improving cognition.

## Supplementary Material

fcag050_Supplementary_Data

## Data Availability

The data of this study are available from the corresponding author on reasonable request. The analysis code generated for this study is provided in the Supplementary material.
