# Supplementary material for: Altered functional connectivity underpins cognitive changes in chronic spinal cord injury
Source: Brain Commun. 2026 Feb 23;8(2):fcag050. doi: 10.1093/braincomms/fcag050 (PMC13098160; doi:10.1093/braincomms/fcag050)
Supplement: fcag050_Supplementary_Data [file fcag050_Supplementary_Data.pdf]

Supplementary material

Sritharan et al. Altered functional connectivity underpins cognitive changes in chronic spinal cord injury

Sample size calculation

The sample size was determined *a priori* using a two-tailed t-test as test family, and "Difference between two independent means (two groups)" as statistical test option with the software G*power[1], given an effect size d=0.811 from a previous verbal fluency study in spinal cord injury (SCI)[2], an error probability α=0.05, a power β=0.8 and equal sample sizes between the two groups. This resulted in a sample size of 25 participants per group, which was further increased to 26 per group.

Test-retest reliability of verbal fluency task

As the verbal fluency task had to be performed silently in the scanner, and then repeated outside the scanner for assessing performance, we determined the test-retest reliability as part of a pilot study.

10 healthy German native speakers (age=29±3.86 years, 8 female) were recruited for the study, who repeated the verbal fluency task after 1 hour. Participants were not told that the same task would be repeated in the second session but instead that there is part 1 and part 2 of a cognitive task. Participants were given 5 letters (S, P, B, M, K) subsequently and performed the phonemic verbal fluency task with one minute per letter according to the Regensburger Wortflüssigkeitstest (RWT).[3] The outcome variable was the sum of correct words across the five letters. Test-retest reliability was assessed by calculating the intraclass correlation coefficient (ICC), using a two-way random-effects model for absolute agreement. Furthermore, a paired t-test was conducted to compare performance between the repetitions.

The ICC for repeating the test after 1 hour was ICC=0.732 (Supplementary Figure 1). According to Koo et al.[4], an ICC less than 0.5 indicates poor reliability, between 0.5 and 0.75 moderate reliability, and between 0.75 and 0.9 good reliability. Therefore, by repeating the verbal fluency task after 1 hour in the main study, it can be ensured that the performance measured outside the magnetic resonance imaging (MRI) scanner reflects the performance inside the scanner with a moderate reliability.

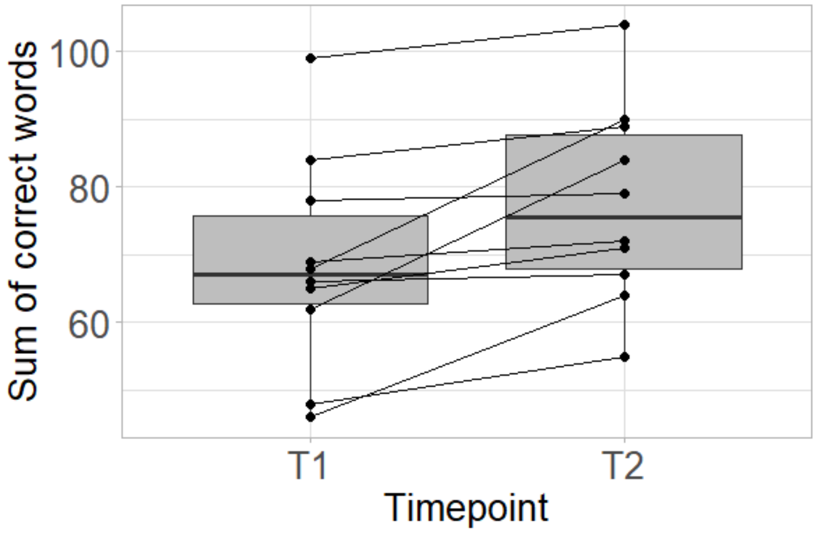

Supplementary Figure 1: Verbal fluency performance assessed during two different timepoints. The verbal fluency task was repeated after one hour in 10 healthy individuals. The lines connect the data of the same individual. Using a two-way random-effects model for absolute agreement, the intraclass correlation coefficient (ICC) was 0.732. Lines connecting two datapoints indicate the performances of the same individual.

In order to evaluate the potential influence of memory effects in the second testing, we determined the average percentage of repeated words (exactly the same word or same root word), which was 28.6%. This means that at most 28.6% of the words may be linked to memory effects. However, we cannot exactly determine, which share of the repeated words were truly produced due to memory effects, and which of the repeated words were produced independently from the first testing session.

Data quality metrics

Below, the data quality metrics generated with the CONN toolbox are being displayed for non-injured controls (Supplementary Figure 2) and SCI individuals (Supplementary Figure 3) separately. In the SCI group, there was one extreme outlier subject across different quality metrics (incl. motion), who was therefore removed from the imaging analysis.

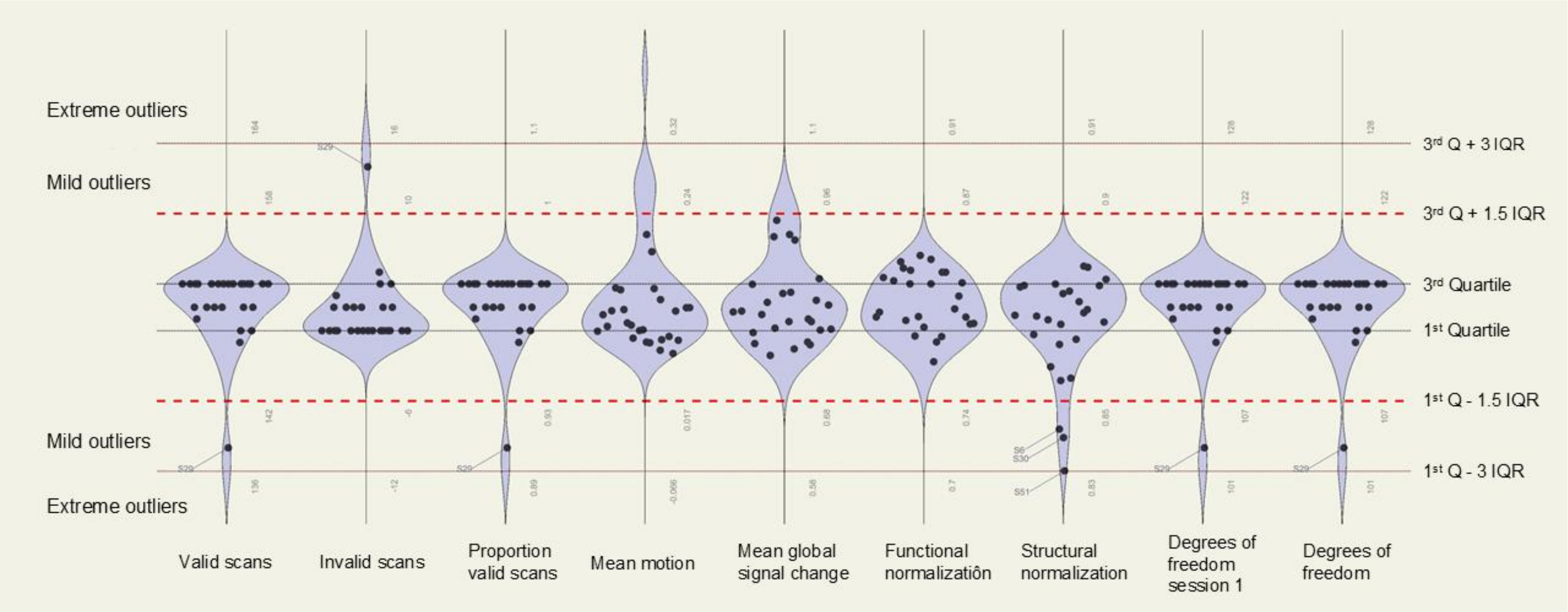

Supplementary Figure 2: Quality control metrics for functional magnetic resonance imaging data in non-injured controls. Single dots represent single subjects (n=26) within the distribution of the respective quality measure. There were no extreme outliers (defined as 3 interquartile ranges (IQR) above the 3rd quartile or below the 1st quartile) for any of the quality measures.

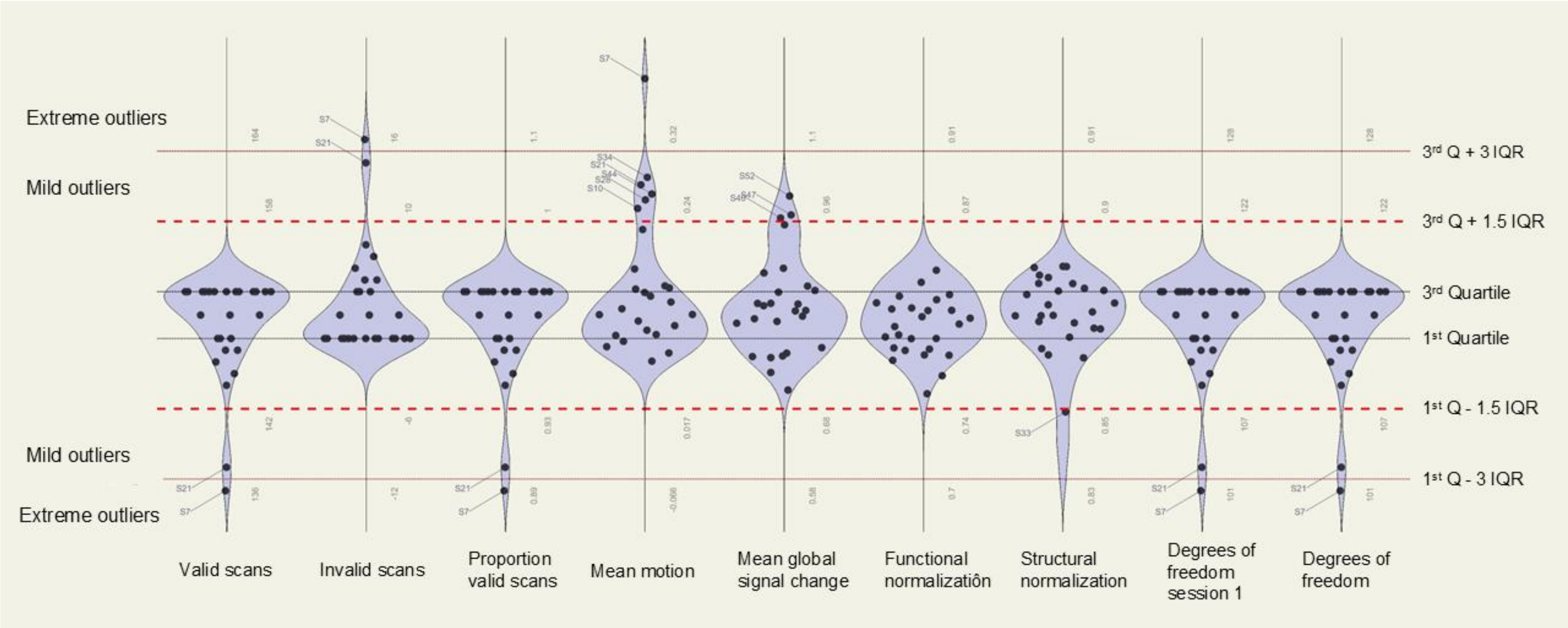

Supplementary Figure 3: Quality control metrics for functional magnetic resonance imaging data in spinal cord injury individuals. Single dots represent single subjects (n=26) within the distribution of the respective quality measure. There was one subject, who was an extreme outlier (defined as 3 interquartile ranges (IQR) above the 3rd quartile or below the 1st quartile) across several quality measures (number of invalid scans, mean motion, degrees of freedom). This subject was therefore discarded from the imaging analysis.

Task activation maps for groups separately

When computing the contrast task vs. rest in both groups (SCI and Non-SCI) individually, the following regions for Non-SCI (Supplementary Table 1, Supplementary Figure 4) and SCI individuals (Supplementary Table 2, Supplementary Figure 5) activated after a family-wise error (FWE) correction with a significance threshold of $p_{FWE}<0.05$ at the cluster-level.

The activated regions in both groups, which are overlapping with the regions in the meta-analysis for phonemic verbal fluency by Wagner et al.[5] and used as seeds in our FC-analysis, are the following: Left inferior frontal gyrus, right insula, right cerebellum and right caudate.

Supplementary Table 1: Activated brain regions during verbal fluency task in the group of non-injured controls.

Region names	Cluster size	P_{FWE}	Peak MNI (x,y,z)
Right cerebellum exterior	1213	<0.001	32, -62, -28
Left supplementary motor cortex	1148	<0.001	-6, 10, 56
Left middle frontal gyrus, left inferior frontal gyrus	2866	<0.001	-46, 22, 26
Right anterior insula, right frontal operculum	230	<0.001	32, 22, 4
Left cerebellum exterior	236	<0.001	-36, -56, -32
Right fusiform gyrus, right inferior occipital gyrus	50	<0.001	28, -92, -8
Left middle frontal gyrus, left precentral gyurs	53	<0.001	-34, 0, 64
Left supramarginal gyrus	18	0.004	-40, -40, 42
Left middle cingulate gyrus	12	0.007	-2, 8, 20
Right thalamus, right caudate	10	0.009	18, -20, 24

Note: FWE: family-wise error corrected, MNI: Montreal Neurological institute

Supplementary Table 2: Activated brain regions during verbal fluency task in spinal cord injury group.

Region names	Cluster size	p_{FWE}	Peak MNI (x,y,z)
Left middle frontal gyrus, left inferior frontal gyrus	2206	<0.001	-42, 24, 22
Right cerebellum exterior	484	<0.001	30, -66, -26
Right anterior insula, right frontal operculum	283	<0.001	32, 26, 0
Left supplementary motor cortex	814	<0.001	-6, 14, 50
Right occipital pole, right fusiform gyrus	39	0.001	24, -94, -6
Left inferior occipital gyrus	46	<0.001	-28, -92, -6
Left caudate	97	<0.001	-16, -2, 20
Left cerebellum exterior	64	<0.001	-30, -58, -28
Right caudate, right thalamus	31	0.001	18, -14, 26
Right precuneus	14	0.006	30, -50, 8

Note: FWE: family-wise error corrected, MNI: Montreal Neurological institute

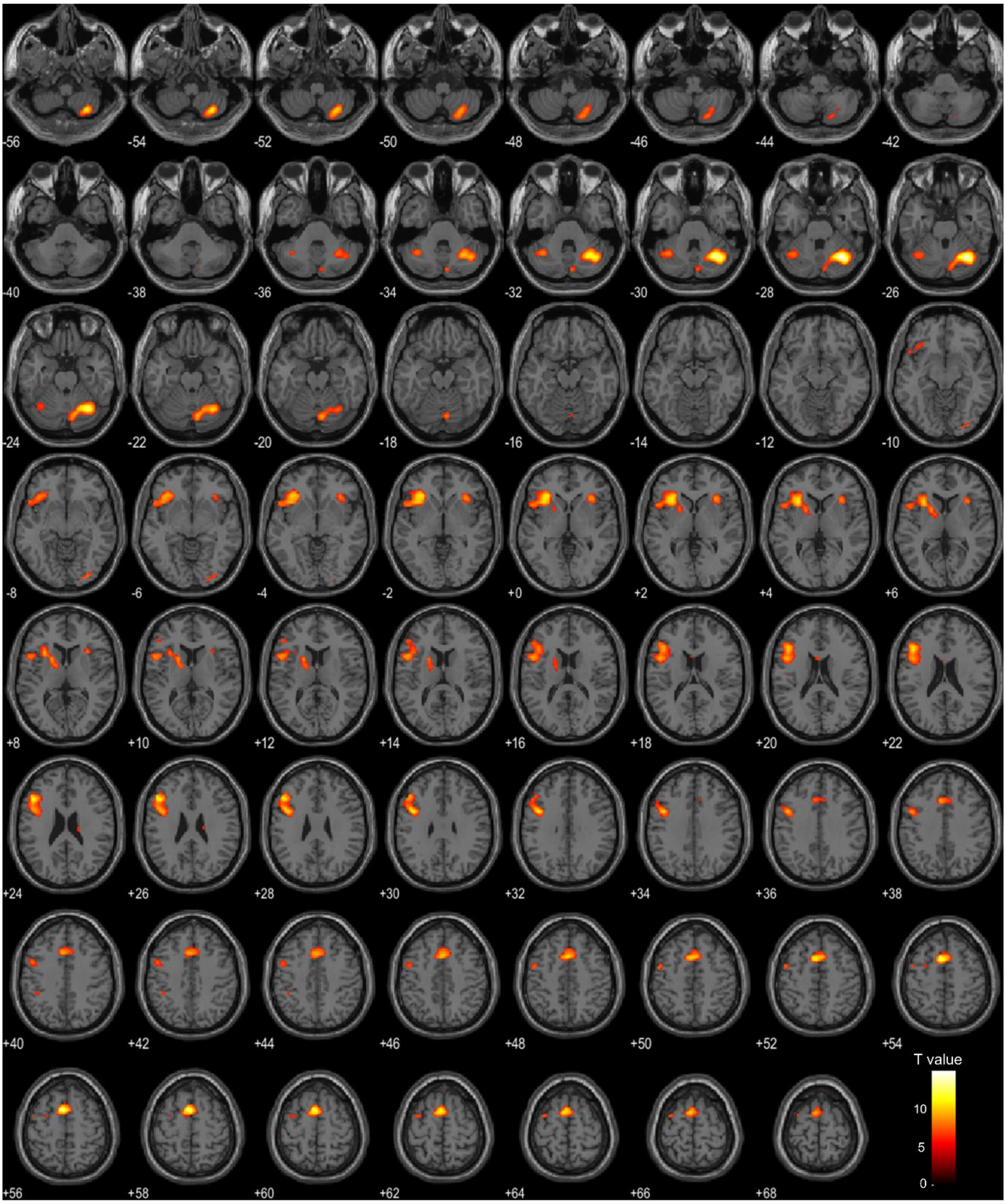

Supplementary Figure 4: Axial slices of activation maps during verbal fluency task in non-injured controls. The heatmap represents T value statistics.

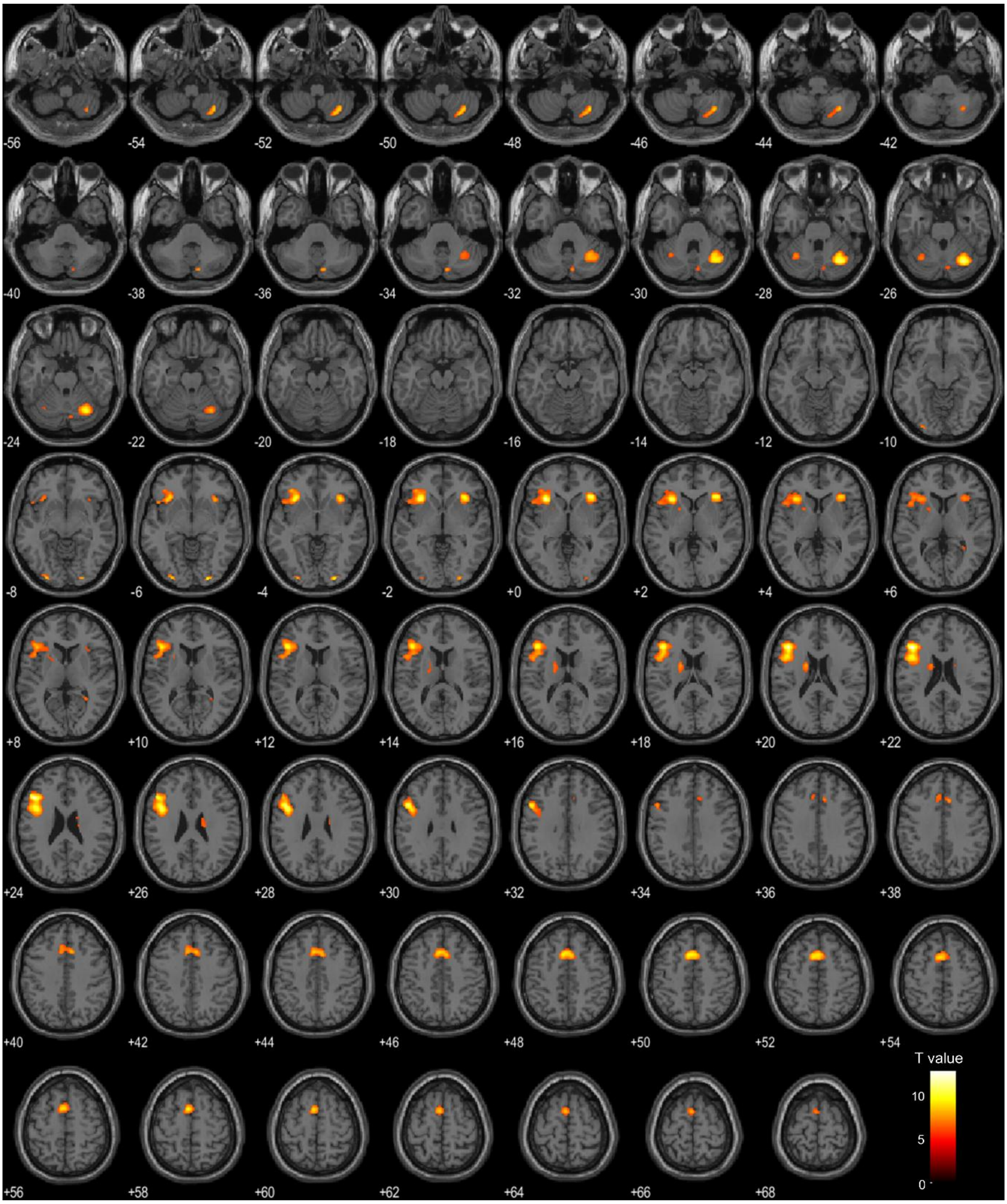

Supplementary Figure 5: Axial slices of activation maps during verbal fluency task in SCI individuals. The heatmap represents T value statistics.

Differences of left and right ASIA scores

Using paired t-tests, differences in the American Spinal Injury Association (ASIA) motor, light touch and pin prick scores between the left and the right side of the body

were compared, in order to investigate differential motor impairment between body sides in the SCI group.

There was a significantly lower motor score in the left side compared to the right side in SCI individuals (t=-2.8, p=0.009) (Supplementary Figure 6A). There was no difference between left and right pin prick and light touch score (Supplementary Figure 6B and 6C).

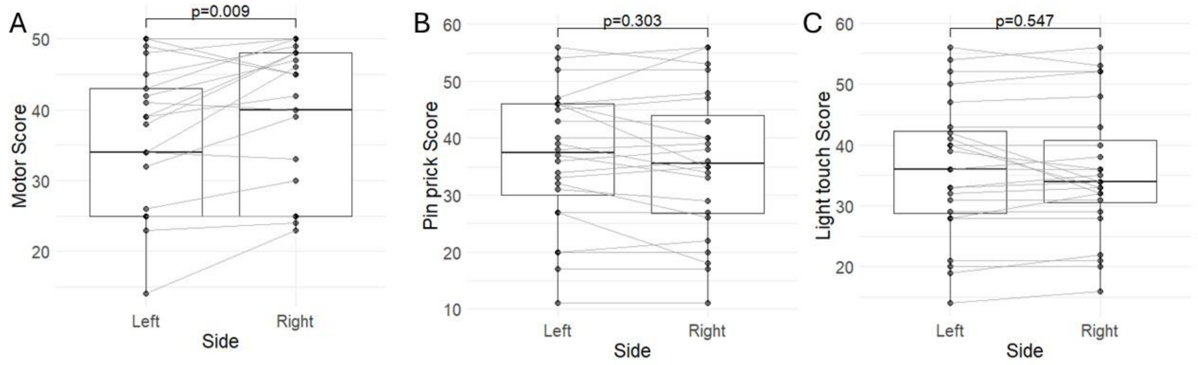

Supplementary Figure 6: Differences between left and right American Spinal Cord Injury Association (ASIA) motor, pin prick and light touch scores in individuals with spinal cord injury (SCI). The boxplots in panel A show a significant difference in the motor score between the left and the right side using a paired t-test (t=-2.8, p=0.009). There was no difference between left and right pin prick score (t=1.05, p=0.303) (panel B) and light touch score (t=0.6, p=0.547) (panel C). Lines connecting two dots indicate the scores from the same individual.

Medication overview

The different pharmaceutical agents taken by the study participants were summarized to several classes of medication, as described below:

- **Bladder medication without antimuscarinics:** Betmiga, Acimethin, Tamsulosin
- **Pain medication without antimuscarinics:** Dafalgan, Pregabalin, Transtec, Venlafaxin
- **Bladder and pain medication with antimuscarinics:** Vesicare, Toviaz, Spasmo-Urgenin, Solifenacin, Mictonorm, Saroten
- **Thyroid:** Euthyrox
- **Birth control:** birth control pill

- **Acne:** Isotretinoin
- **Cholersterol:** Atorvastatin
- **Blood pressure:** Lisinopril, Exforge, Indapamide, Metropolol
- **Human immunodeficiency virus:** Truvada
- **Spasticity:** Lioresal, Sirdalud, Baclofen
- **Constipation:** Movicol, Lecicarbon
- **Rheuma:** Arcoxia

Supplementary Table 3: Demographic and clinical characteristics of the spinal cord injury participants

ID	Age	Sex	Education level	Handed-ness	TSI [years]	Lesion level	Etiology	Current AIS level	Initial AIS level	Rehabilitation span [months]
1	34	female	College of higher education	Right	9	L1	Sports accident	B	B	7
2	56	male	Apprenticeship	Left	3	C2	Accident	D	D	2
3	50	male	Grammar school	Left	30	Th12	NA	A	A	4
4	50	male	University	Right	4	C6	Bike accident	D	D	9
5	32	male	University	Right	9	C4	Accident	D	D	7
6	32	female	Compulsory school	Right	12	Th6	Bike accident	A	A	5
7	54	female	Apprenticeship	Right	23	Th12	Vehicle accident	A	A	5
8	58	male	University	Right	6	Th4	Bike accident	A	A	7
9	59	male	Apprenticeship	Right	5	C3	Sports accident	D	D	6
10	37	male	University	Right	30	Th12	NA	D	D	5
11	55	male	Apprenticeship	Right	29	C6	Vehicle accident	D	D	4
12	57	female	University	Right	40	C7	Sports accident	D	D	NA
13	43	male	College of higher education	Right	21	C5	Accident	A	A	7

14	25	male	College of higher education	Right	6	C7	Fall	D	D	8
15	34	male	College of higher education	Right	15	Th12	Vehicle accident	A	A	6
16	46	female	University	Right	4	Th10	Sports accident	A	A	6
17	49	male	University	Right	2	C5	Sports accident	A	A	10
18	38	male	Grammar school	Left	2	C3	Sports accident	D	D	4
19	47	female	Apprenticeship	Right	6	Th5	Vehicle accident	C	B	7
20	24	male	Apprenticeship	Right	1	Th8	Vehicle accident	A	A	4
21	55	male	Apprenticeship	Left	12	C5	Vehicle accident	D	D	3
22	60	male	Apprenticeship	Left	8	Th12	Accident	D	D	6
23	57	male	Apprenticeship	Right	17	Th3	Accident	A	A	NA
24	37	female	Apprenticeship	Right	18	Th4	Vehicle accident	A	A	6
25	47	male	College of higher education	Right	30	Th3	Vehicle accident	C	C	8
26	55	female	Grammar school	Right	3	C5	Fall	D	C	NA

Note: NA: not available, TSI: time since injury

Age- and education-adjusted percentile ranks of verbal fluency performance

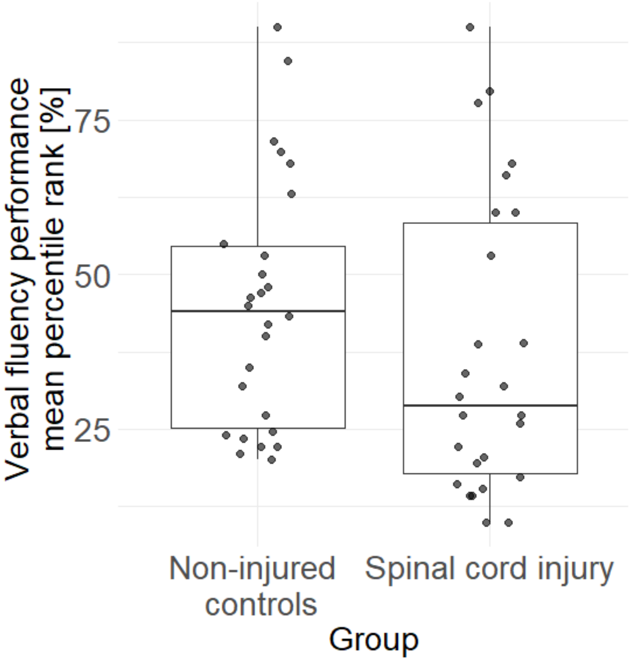

Supplementary Figure 7: Visualization of age- and education-adjusted percentile ranks of verbal fluency performance in individuals with (n=26) and without spinal cord injury (n=26). The boxplot shows the mean percentile rank for the five letters of the Regensburger Wortflüssigkeitstest (RWT) for each individual, indicating the percentage of people from the norm population of the RWT in the same age and education group, who performed equally or worse. Single datapoints indicate the percentile rank of single individuals.

Correlation of verbal fluency performance with brain activity in all participants

We studied whether performance in verbal fluency correlated with the blood-oxygen-level dependent (BOLD) signal in all study participants. Beta-values were extracted from regions which showed significant group differences between spinal cord injury and non-injured controls, and partial Pearson correlation was applied to correlate those beta-values with verbal fluency performance, using age as covariate.

Higher beta-values from the significant cluster with peak in the right putamen were associated with higher verbal fluency performance in all subjects (r=0.324, p=0.022) (Supplementary Figure 8).

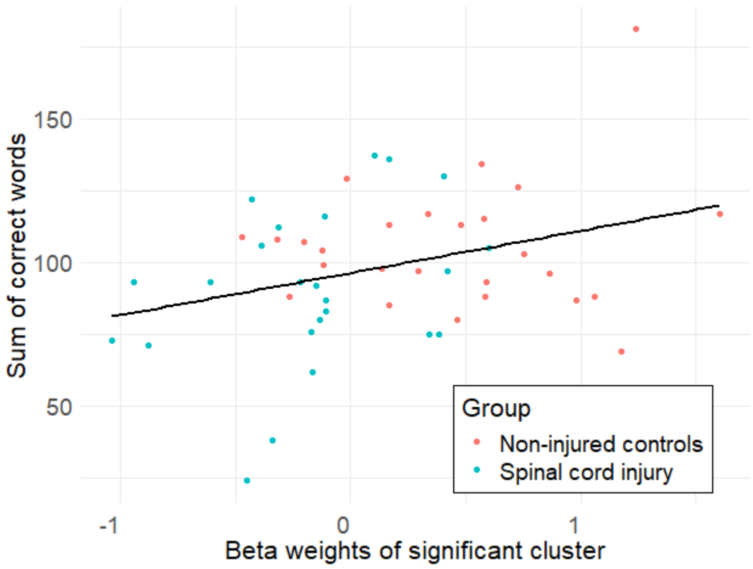

Supplementary Figure 8: Correlation of verbal fluency performance with beta weights of significant cluster using partial Pearson correlation with age as covariate. The scatterplot shows the correlation between beta weights from the significant cluster with peak in the right putamen, extracted from the general linear model comparing the blood-oxygen-level dependent signal between the two groups, and the sum of correct words across all subjects (r=0.324, p=0.022). Orange datapoints represent non-injured controls and blue datapoints depict individuals with spinal cord injury.

Subgroup analysis between AIS A and D

For the subgroup analysis, we repeated the analyses described in the methods section of the manuscript for comparing 11 individuals with American Spinal Cord injury Association (ASIA) impairment scale (AIS) A with 12 individuals with AIS D. The demographic and clinical characteristics are described in Supplementary Table 4. The two groups did not significantly differ in any of the variables, except for the International Standards for Neurological Classification of spinal cord injury (ISNCSCI) total motor score and the level of injury (Supplementary Table 4).

Supplementary Table 4: Clinical and demographic characteristics of individuals with spinal cord injury with AIS A and AIS D.

	AIS A (n=11)	AIS D (n=12)	Test statistics	p-value
Sex (male/female)	7/4	10/2	χ^2=1.2	0.28
Age [Years, mean±SD]	44±11.1	48.3±11.9	W=47	0.25
Time since injury [Years, mean±SD]	13.5±9.4	12.6±12.9	W=73.5	0.67
NPRS current pain [mean±SD]	2.6±2.0	2.7 ±2.5	t=-0.1	0.90
STAI anxiety state [mean±SD]	36.1±9.1	38.92±6.5	t=43.5	0.17
STAI anxiety trait [mean±SD]	36.45±8.5	37±9.7	t=-0.1	0.89
Level of injury				
C1-C4	0 (0%)	4 (33.3%)	χ^2=10.4	**0.005**
C5-C8	2 (18.2%)	6 (50%)		
T1-S5	9 (81.8%)	2 (16.7%)		
ISNCSCI				
Total motor score [mean±SD]	53.4±16.9	89.8±6.9	W=11.5	**0.001**
Total light touch score [mean±SD]	60.9±23.0	77.5±18.8	W=36	0.12
Total pinprick score [mean±SD]	59.2±25.1	80.3±20.7	W=29.5	0.05
Medication n (%)				
Bladder without antimuscarinics	5 (45.5%)	1 (8.3%)	χ^2=2.4	0.12
Antimuscarinics	5 (45.5%)	4 (33.3%)	χ^2=0.03	0.87
Pain	5 (45.5%)	4 (33.3%)	χ^2=0.03	0.87
High blood pressure	1 (9.1%)	4 (33.3%)	χ^2=0.8	0.37
Spasticity	1 (9.1%)	3 (25%)	χ^2=0.2	0.65
Others (constipation, antibiotics, rheumatism, cholesterol)	2 (18.2%)	3 (25%)	χ^2<0.01	1

Note: AIS: American Spinal Cord injury Association (ASIA) impairment scale, ISNCSCI: International Standards for Neurological Classification of spinal cord injury, NA: Not applicable, NPRS: numeric pain rating scale, SD: standard deviation, STAI: state-trait anxiety inventory

On average, the sum of correct words was 92.27±25.36 in the AIS A and 86.67±32 in the AIS D group. The two groups did not significantly differ in the sum of correct words

(W=78.5, p=0.46) (Supplementary Figure 9A). There was no significant difference in the number of below-average performances between AIS A and AIS D (W=63, p=0.874) (Supplementary Figure 9B). Neither were there any differences between the two groups with respect to brain activity or seed-based functional connectivity during the verbal fluency task.

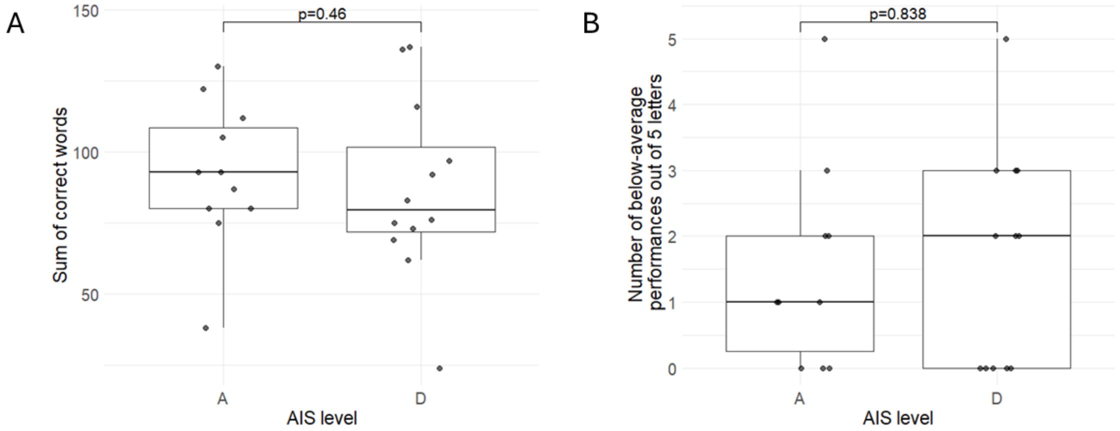

Supplementary Figure 9: Verbal fluency performance in individuals with spinal cord injury (SCI) with American Spinal Cord Injury Association (ASIA) impairment scale (AIS) A and AIS D. The boxplots in panel A show no significant difference in the sum of correct words between the AIS A (N=11) and the AIS D (N=12) group using a Wilcoxon rank sum test (W=78.5, p=0.46). Neither is there a significant difference in the number of below-average performances (ranging from 0 to 5) between the two groups (W=63, p=0.874).

Subgroup analysis between cervical and thoracic SCI

There were 13 individuals with thoracic and 12 individuals with cervical SCI. The individuals did not differ in any of the demographic and clinical variables, except for the ISNCSCI total motor score (see Supplementary Table 5).

Supplementary Table 5: Clinical and demographic characteristics of individuals with spinal cord injury with cervical and thoracic SCI.

	Cervical SCI (n=12)	Thoracic SCI (n=13)	Test statistics	p-value
Sex (male/female)	10/2	8/5	χ^2=1.5	0.23
Age [Years, mean±SD]	47.8±11	44.8±11.2	W=91.5	0.48
Time since injury [Years, mean±SD]	11.3±12.3	15.4±10.4	W=53	0.18
NPRS current pain [mean±SD]	2.67±2.5	2.69±2.1	t=-0.03	0.98
STAI anxiety state [mean±SD]	38.8±6.7	36.3±9.3	t=0.78	0.44
STAI anxiety trait [mean±SD]	37.5±8.2	36.8±9.7	t=-0.2	0.86
ISNCSCI				
Total motor score [mean±SD]	84.6±17.1	62.2±19.7	W=108.5	**0.03**
Total light touch score [mean±SD]	68.1±18.9	73.3±24.2	W=63	0.64
Total pinprick score [mean±SD]	71.4±25.1	71.8±24.2	W=73.5	0.93

Note: AIS: American Spinal Cord injury Association (ASIA) impairment scale, ISNCSCI: International Standards for Neurological Classification of spinal cord injury, NPRS: numeric pain rating scale, SD: standard deviation, STAI: state-trait anxiety inventory

When comparing performance on the verbal fluency task, there was no significant difference in the sum of correct words between the two groups (t=1.265, p=0.219) (Supplementary Figure 10A). Neither was there a significant difference between the number of below-average performances between individuals with cervical compared with thoracic SCI (W=71, p=0.714) (Supplementary Figure 10B).

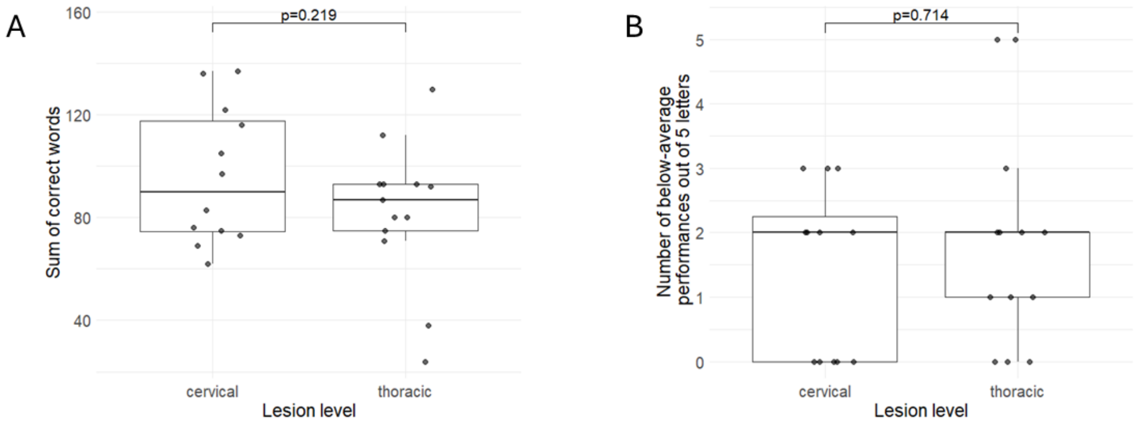

Supplementary Figure 10: Boxplots showing differences in verbal fluency performance between individuals with cervical and thoracic spinal cord injury (SCI). Single datapoints indicate the performance of individual participants, and the bold horizontal lines in the boxplots indicate the median within each group. Panel A shows no difference in the sum of correct words using a t-test (t=1.265, p=0.219) in individuals with cervical SCI (N=12) compared to the thoracic SCI (N=13). The boxplot in panel B shows the number of below-average performances (ranging from 0 to 5) in each of the groups. There was no significant difference in the number of below-average performances between cervical and thoracic SCI using a Wilcoxon rank sum test (W=71, p=0.714).

With respect to the comparison of the BOLD signal between individuals with cervical and thoracic SCI, there were no significant differences in the brain. Neither were there any significant differences in seed-based functional connectivity between the two groups.

Subgroup analysis with antimuscarinics intake

There were 15 SCI individuals who did not take any medication containing antimuscarinics and 11 who were taking antimuscarinics. The two groups did not differ in any of the clinical or demographic variables except for the time since injury (Supplementary Table 6).

Supplementary Table 6: Clinical and demographic characteristics of individuals with spinal cord injury taking/not taking medications containing antimuscarinics.

	SCI with antimuscarinics (n=11)	SCI without antimuscarinics (n=15)	Test statistics	p-value
Sex (male/female)	7/4	11/4	χ^2=0.3	0.60
Age [Years, mean±SD]	45.3±12.0	46.2±10.6	t=0.21	0.837
Time since injury [Years, mean±SD]	8.1±6.3	17.1±12.4	t=2.19	**0.039**
NPRS current pain [mean±SD]	2.9±2.2	2.5±2.3	t=-0.50	0.624
STAI anxiety state [mean±SD]	37.1±6.5	37.7±9.1	t=0.20	0.844
STAI anxiety trait [mean±SD]	36.9±9.5	37.4±8.2	t=0.14	0.889
Level of injury				
C1-C4	1 (9.1%)	3 (20%)	χ^2=0.9	0.636
C5-C8	3 (27.3 %)	5 (33.3%)		
T1-S5	7 (63.6%)	7 (46.7%)		
ISNCSCI				
Total motor score [mean±SD]	66.7±20.9	76.6±20.9	W=99.5	0.223
Total light touch score [mean±SD]	65.8±24.1	72.2±23.3	t=0.68	0.503
Total pinprick score [mean±SD]	68.6±29.1	71.1±23.8	t=0.23	0.817

Note: AIS: American Spinal Cord injury Association (ASIA) impairment scale, ISNCSCI: International Standards for Neurological Classification of spinal cord injury, NPRS: numeric pain rating scale, SD: standard deviation, STAI: state-trait anxiety inventory

There was no significant difference in the sum of correct words between individuals taking antimuscarinics compared to individuals not taking antimuscarinics (t=0.65, p=0.52) (Supplementary Figure 11A). Neither was there a significant difference in the number of below-average performances (W=88.5, p=0.768) (Supplementary Figure 11B).

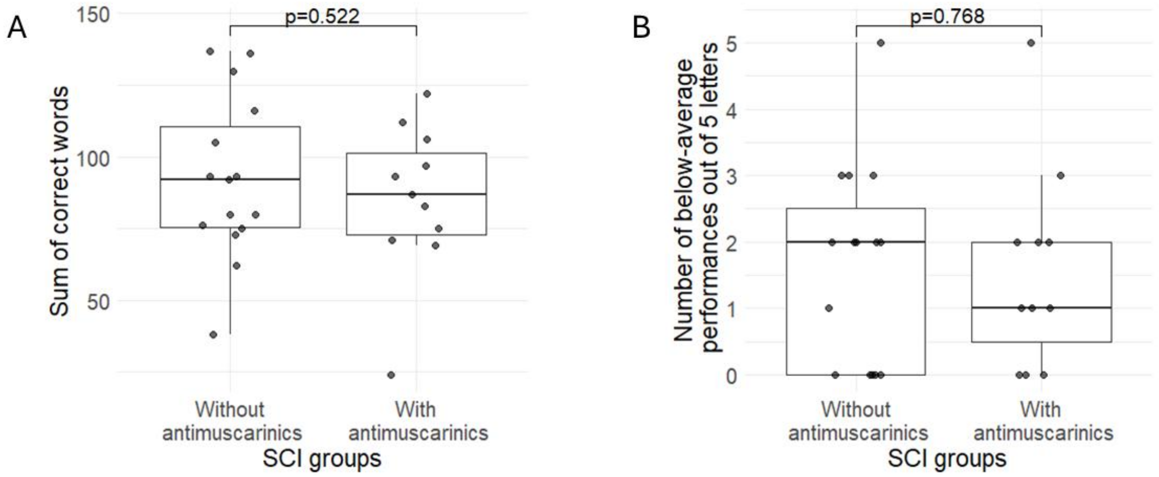

Supplementary Figure 11: Boxplots showing differences in verbal fluency performance between spinal cord injury (SCI) individuals taking/not taking medication containing antimuscarinics. Single datapoints indicate the performance of individual participants, and the bold horizontal lines in the boxplots indicate the median within each group. Panel A shows no difference in the sum of correct words using a t-test (t=0.65, p=0.52) in individuals taking antimuscarinics (N=11) compared to individuals not taking antimuscarinics (N=15). The boxplot in panel B shows the number of below-average performances (ranging from 0 to 5) in each of the groups. There was no significant difference in the number of below-average performances between individuals taking and not taking antimuscarinics using a Wilcoxon rank sum test (W=88.5, p=0.768).

With respect to the comparison of the BOLD signal between SCI individuals taking vs. not taking antimuscarinics, there were no significant differences in the brain activity, nor in seed-based functional connectivity.

Regression model including medication as independent variable

As different types of medication taken by individuals with SCI may have an adverse effect on cognition, in particular medication including antimuscarinics[6], a linear regression model was implemented. Specifically, the sum of correct words in the verbal fluency task was included as main outcome variable, and predicted based on: i) group, ii) intake of pain medication without antimuscarinics, iii) medication with

antimuscarinics and iv) medication against spasticity (all binary variables). None of the included predictors showed a significant effect on verbal fluency performance. The detailed parameters of the regression model are displayed in Supplementary Table 7 below. Out of the four predictors, group showed the strongest influence on verbal fluency performance, as it had the highest absolute value in the estimate and the t-value.

Supplementary Table 7: Parameters of the regression model including medication intake.

Variable	Estimate	t-value	p-value
Group (SCI/Non-SCI)	-12.058	-1.385	0.173
Antimuscarinics (Yes/No)	-2.788	-0.223	0.824
Pain medication without antimuscarinics (Yes/No)	-4.051	-0.367	0.715
Spasticity (Yes/No)	-6.051	-0.386	0.701

However, this analysis holds some limitations: Firstly, the model gained complexity by adding several variables as predictors, therefore reducing the degrees of freedom and potentially the statistical power.[7] Secondly, it cannot be ruled out whether there is truly no effect of medication on the verbal fluency performance, as the sample size might be too small for showing an effect of medication. Furthermore, the predicting variables are not truly independent from each other, as medication intake in the SCI group is generally higher compared to the Non-SCI group.

Subgroup analysis with pain medication intake

15 individuals with SCI did not take any pain medication, whereas 11 were on pain medication at the timepoint of the study. Pain medication included Dafalgan, Pregabalin, Transtec, Venlafaxin and Saroten. The two groups did not differ in any of the demographical and clinical variables except for time since injury and the state-trait anxiety inventory (STAI) state score (Supplementary Table 8).

Supplementary Table 8: Clinical and demographic characteristics of individuals with spinal cord injury taking/not taking pain medications.

	SCI with pain medication (n=11)	SCI without pain medication (n=15)	Test statistics	p-value
Sex (male/female)	6/5	12/3	χ^2=1.93	0.16
Age [Years, mean±SD]	46.5±10.5	45.3±11.7	W=81.5	0.98
Time since injury [Years, mean±SD]	5.7±6.2	18.8±10.7	W=148	**<0.001**
NPRS current pain [mean±SD]	3.5±2.3	2.1±2	t=-1.59	0.13
STAI anxiety state [mean±SD]	41.9±8.4	34.2±6	t=-2.6	**0.02**
STAI anxiety trait [mean±SD]	39.6±10	35.4±7.2	t=-1.2	0.25
ISNCSCI				
Total motor score [mean±SD]	70.5±20.3	73.4±22.2	W=108.5	0.76
Total light touch score [mean±SD]	65.3±20.8	72.6±25.3	W=63	0.47
Total pinprick score [mean±SD]	68.2±25.7	71.4±26.4	W=73.5	0.93

Note: AIS: American Spinal Cord injury Association (ASIA) impairment scale, ISNCSCI: International Standards for Neurological Classification of spinal cord injury, NPRS: numeric pain rating scale, SD: standard deviation, STAI: state-trait anxiety inventory

There was no significant difference in the sum of correct words between individuals taking pain medication compared to individuals not taking pain medication (t=0.648, p=0.523) (Supplementary Figure 12A). Neither was there a significant difference in the number of below-average performances (W=70.5, p=0.537) (Supplementary Figure 12B).

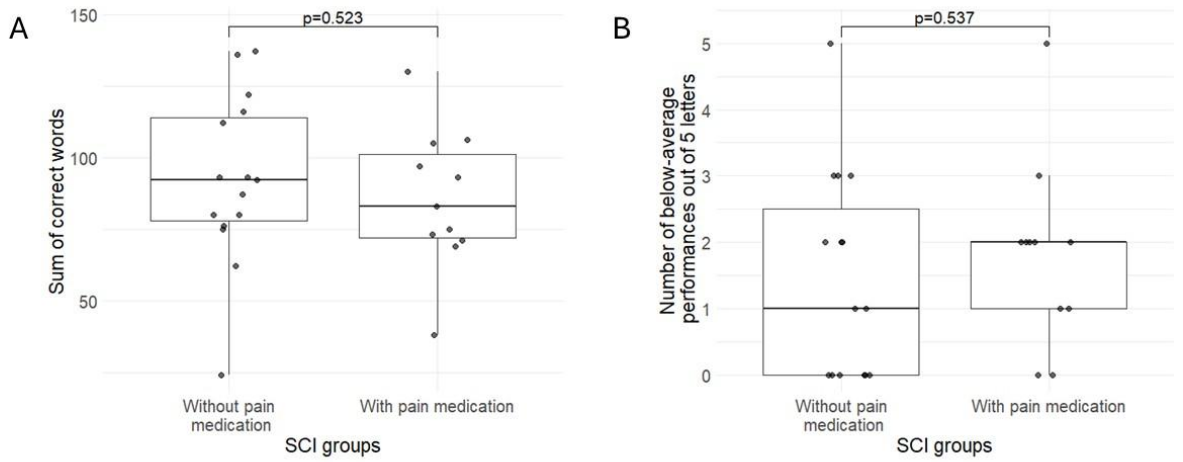

Supplementary Figure 12: Boxplots showing differences in verbal fluency performance between spinal cord injury (SCI) individuals taking/not taking pain medication. Single datapoints indicate the performance of individual participants, and the bold horizontal lines in the boxplots indicate the median within each group. Panel A shows no difference in the sum of correct words using a t-test (t=0.648, p=0.523) in individuals taking pain medication (N=11) compared to individuals not taking pain medication (N=15). The boxplot in panel B shows the number of below-average performances (ranging from 0 to 5) in each of the groups. There was no significant difference in the number of below-average performances between individuals taking and not taking pain medication using a Wilcoxon rank sum test (W=70.5, p=0.537).

When comparing the BOLD signal during the verbal fluency task in SCI individuals taking pain medication vs. not taking pain medication, no significant differences were observed.

When performing a group comparison in seed-based functional connectivity, we found significantly decreased functional connectivity between the left inferior frontal gyrus (seed) and the precuneus ($p_{FWE\text{-}Bonferroni\text{-}corrected\ (BF)}$=0.0003, cluster size=335 voxels, peak MNI coordinates=-8, -68, 52) in individuals not taking pain medication, compared to those taking pain medication (Supplementary Figure 13).

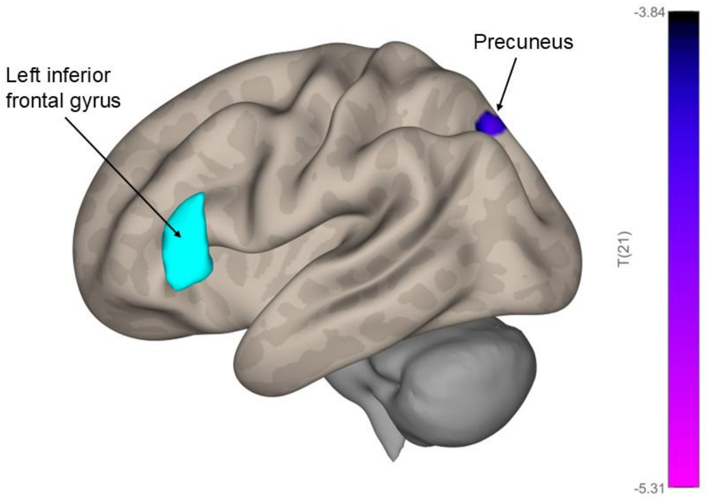

Supplementary Figure 13: Group comparison in functional connectivity between individuals with spinal cord injury taking vs. not taking pain medication using a general linear model. There was significantly altered functional connectivity (p=0.0003, corrected with family-wise error and Bonferroni, cluster size=335 voxels) between the left inferior frontal gyrus (seed) and a cluster containing the precuneus between SCI individuals taking pain medication (N=11) and individuals not taking pain medication (N=15).

Code for analysis in R

```r
#Verbal fluency analysis - Altered functional connectivity underpins cognitive changes in chronic spinal cord injury


#Load packages

library(readxl)

library(rstatix)

library(ggplot2)

library(dplyr)

library(lme4)

library(lmerTest)

library(car)

library(stats)

library(ggsignif)

library(ppcor)

library(R.matlab)

library(npsm)

library(stringr)

library(tidyr)


# Data import ------------------------------------------------------------

VF_behav_excel <- read_excel("D:/Main_study/Verbal_fluency_results.xlsx") #contains the performances in the verbal fluency ta
sk per subject

VF_behav <- as.data.frame(VF_behav_excel)

VF_behav$Group <- as.factor(VF_behav$Group)

VF_behav$Education <- factor(VF_behav$Education,levels=c("Obligatorische_Schule","Berufslehre" ,"Mittelschule","Hoehere_Beruf
sbildung","Universitaet")) #define levels in this order

VF_behav$Sex <- as.factor(VF_behav$Sex)

VF_behav$ASIA_score <- as.factor(VF_behav$ASIA_score)

VF_behav$Pain_medication <- as.factor(VF_behav$Pain_medication)

VF_behav$Pain_without_antimuscarinics <- as.factor(VF_behav$Pain_without_antimuscarinics)

VF_behav$Antimuscarinics <- as.factor(VF_behav$Antimuscarinics)

VF_behav$Spasticity <- as.factor(VF_behav$Spasticity)

VF_behav$Mean_Correctwords <- rowMeans(VF_behav[,c("Correctwords_B","Correctwords_G","Correctwords_K","Correctwords_M","Corre
ctwords_P","Correctwords_R","Correctwords_S")],na.rm=TRUE)


#Import scores from INSCSCI examination

ASIA_results <- read_excel("D:/Main_study/SCI-Cog_INSCSCI-results_left_right.xlsx")

ASIA_results <- as.data.frame(ASIA_results)


# Evaluate questionnaire data ---------------------------------------------

#Questionnaire data (e.g. NRS, STAI, SWLS)

data_SCICO <- read_excel("D:/Main_study/Secutrial_Questionnaire_answers_conv.xlsx")

df_secutrial <- data_SCICO[data_SCICO$mnpaid %in% VF_behav$ID, ]  #Only keep the rows of valid subjects

colnames(df_secutrial)[5] <- "Group" #rename group column

df_secutrial$Group[df_secutrial$Group=='HC'] <- "Non-SCI"

df_secutrial$Group <- as.factor(df_secutrial$Group)

df_secutrial$Sum_correct_words <- VF_behav$Sum_Correctwords


# NRS: Pain -------------------------------------------------------------------

#Calculate mean and standard deviation
```

```
df_secutrial %>%
  group_by(Group) %>%
  summarise(current_mean_pain = mean(v12166_1_schmerzlevel_intpain, na.rm = TRUE))

df_secutrial %>%
  group_by(Group) %>%
  summarise(sd_mean_pain = sd(v12166_1_schmerzlevel_intpain, na.rm = TRUE))

pain_summary <- df_secutrial %>%
  group_by(Group) %>%
  summarise(
    mean_pain = mean(v12166_1_schmerzlevel_intpain),
    se_pain = sd(v12166_1_schmerzlevel_intpain) / sqrt(n())
  )

#Test for difference in pain level between the two groups
wilcox.test(v12166_1_schmerzlevel_intpain~Group,data=df_secutrial)

# STAI: Anxiety  ---------------------------------------------------------------------
cols_stai_state_scale <- colnames(df_secutrial)[72:91]  #state scale
cols_stai_trait_scale <- colnames(df_secutrial)[92:111] #trait scale

#Replace the character answers into their numeric corresponding answers
df_secutrial <- df_secutrial %>%
  mutate(across(all_of(cols_stai_state_scale), ~ case_when(
    . =="ueberhaut nicht" ~ "1",
    . == "ein wenig" ~ "2",
    . == "ziemlich" ~ "3",
    . == "sehr" ~ "4",

    TRUE ~ .  # Keep other values unchanged
  )))

df_secutrial <- df_secutrial %>%
  mutate(across(all_of(cols_stai_trait_scale), ~ case_when(
    . =="fast nie" ~ "1",
    . == "manchmal" ~ "2",
    . == "oft" ~ "3",
    . == "fast immer" ~ "4",

    TRUE ~ .  # Keep other values unaltered
  )))

#convert character into numeric columns
df_secutrial[, c(72:111)] <- lapply(df_secutrial[, c(72:111)], as.numeric)

#Invert following items of the questionnaire according to https://diabetes-psychologie.de/downloads/Beschreibung_STAI.pdf
df_secutrial <- df_secutrial %>%
  mutate_at(vars(72,73,76,79,81,82,86,87,90,91,92,97,98,101,104,107,110), ~ 5 - .) #subtract current column value from 5 to t
urn a 1 -> 4, 2 -> 3, 3 -> 2, 4 -> 1
```

```
#Build sum per scale per person
df_secutrial$sum_stai_state <- rowSums(df_secutrial[,cols_stai_state_scale])
df_secutrial$sum_stai_trait <- rowSums(df_secutrial[,cols_stai_trait_scale])

#Check normality and equal variance assumption for t-test
leveneTest(df_secutrial$sum_stai_trait, df_secutrial$Group)  #homogeneity of variances is not fulfilled
by(df_secutrial$sum_stai_state,df_secutrial$Group, shapiro.test) #not fulfilled

leveneTest(df_secutrial$sum_stai_state, df_secutrial$Group)  #homogeneity of variances is fulfilled
by(df_secutrial$sum_stai_state,df_secutrial$Group, shapiro.test) #not fulfilled

wilcox.test(sum_stai_state~Group,data=df_secutrial)
wilcox.test(sum_stai_trait~Group,data=df_secutrial)

#Append NRS and STAI values to verbal fluency dataframe
names(df_secutrial)[names(df_secutrial) == "mnpaid"] <- "ID"

VF_behav <- VF_behav %>%
  left_join(df_secutrial %>% dplyr::select(ID, sum_stai_trait, sum_stai_state, v12166_1_schmerzlevel_intpain),
            by = "ID")

names(VF_behav)[names(VF_behav) == "v12166_1_schmerzlevel_intpain"] <- "pain"



# Decode demographic and clinical vectors from CONN -----------------------
sex_male_vector <- as.numeric(VF_behav$Sex)-1
sex_female_vector <- (as.numeric(VF_behav$Sex)-2)*(-1)
group_SCI_vector <- as.numeric(VF_behav$Group)-1
group_nonSCI_vector <- (as.numeric(VF_behav$Group)-2)*(-1)

TSI_vec <- VF_behav$TSI
TSI_vec[is.na(TSI_vec)] = 0

pain_medication_vec <- as.numeric(VF_behav$Pain_medication)-1
pain_medication_vec <- pain_medication_vec*group_SCI_vector
nopain_medication_vec <- (as.numeric(VF_behav$Pain_medication)-2)*(-1)
nopain_medication_vec <- nopain_medication_vec*group_SCI_vector

age_SCI_painmedication <- VF_behav$Age*pain_medication_vec
age_SCI_nopainmedication <- VF_behav$Age*nopain_medication_vec

VF_painmedication <- VF_behav$Sum_Correctwords*pain_medication_vec
VF_nopainmedication <- VF_behav$Sum_Correctwords*nopain_medication_vec

antimuscarinics_vec <- as.numeric(VF_behav$Antimuscarinics)-1
antimuscarinics_vec <- antimuscarinics_vec*group_SCI_vector
no_antimuscarinics_vec <- (as.numeric(VF_behav$Antimuscarinics)-2)*(-1)
no_antimuscarinics_vec <- no_antimuscarinics_vec*group_SCI_vector

VF_behav_patientonly <- VF_behav[VF_behav$Group == 'SCI',]
```

```r
VF_behav_controlsonly <- VF_behav[VF_behav$Group == 'Non-SCI',]


# Demographic and clinical characteristics --------------------------------

mean(VF_behav$Age[VF_behav$Group=="SCI"])

mean(VF_behav$Age[VF_behav$Group=="Non-SCI"])

sd(VF_behav$Age[VF_behav$Group=="SCI"])

sd(VF_behav$Age[VF_behav$Group=="Non-SCI"])

mean(VF_behav$TSI[VF_behav$Group=="SCI"])

sd(VF_behav$TSI[VF_behav$Group=="SCI"])


#Test for difference of age between the two groups

wilcox_test(Age~Group,data=VF_behav)


#Test for difference of Sex between the two groups

table(VF_behav$Group, VF_behav$Sex)

chisq.test(VF_behav$Sex,VF_behav$Group,correct=FALSE)


#Difference in handedness

contingency_table <- data.frame("SCI"=c(21,5),"Non-SCI"=c(25,1),row.names=c("Right","Left"))

chisq.test(contingency_table)


# Calculate Chi square test if there are significant differences in education level between the groups

aggregate(VF_behav$Education,by=list(VF_behav$Group,VF_behav$Education),FUN=length)

contingency_table <- data.frame("SCI"=c(1,9,4,5,7),"Non-SCI"=c(0,4,2,4,16),row.names=c("Obligatorische_Schule","Berufslehre",
"Mittelschule","Hoehere_Berufsbildung","Universitaet"))

mosaicplot(contingency_table,color=TRUE)

chisq.test(contingency_table) #There is no significant difference in education level between the groups


#Difference in medication intake using Chi square test

bladder_med_table <- matrix(c(9,17,0,26),nrow=2,byrow=TRUE) #order SCI took med, SCI did not take med, HC took med, HC did no
t take med

chisq.test(bladder_med_table)


antimuscarinics_med_table <- matrix(c(11,15,0,26),nrow=2,byrow=TRUE) #order SCI took med, SCI did not take med, HC took med,
HC did not take med

chisq.test(antimuscarinics_med_table)


pain_med_table <- matrix(c(11,15,1,25),nrow=2,byrow=TRUE) #order SCI took med, SCI did not take med, HC took med, HC did not
take med

chisq.test(pain_med_table)


bloodpressure_med_table <- matrix(c(5,21,1,25),nrow=2,byrow=TRUE) #order SCI took med, SCI did not take med, HC took med, HC
did not take med

chisq.test(bloodpressure_med_table)


spasticity_med_table <- matrix(c(5,21,0,26),nrow=2,byrow=TRUE) #order SCI took med, SCI did not take med, HC took med, HC did
not take med

chisq.test(spasticity_med_table)


constipation_med_table <- matrix(c(2,24,0,26),nrow=2,byrow=TRUE) #order SCI took med, SCI did not take med, HC took med, HC d
id not take med

chisq.test(constipation_med_table)
```

```r
others_med_table <- matrix(c(5,21,3,23),nrow=2,byrow=TRUE) #order SCI took med, SCI did not take med, HC took med, HC did not take med

chisq.test(others_med_table)


contingency_table <- data.frame("SCI"=c(9,11,11,5,5,7),"Non-SCI"=c(0,0,1,1,0,3),row.names=c("Bladder","antimuscarinics","pain","blood_pressure","spasticity","others"))

mosaicplot(contingency_table,color=TRUE)

chisq.test(contingency_table)


#Create histogram with ASIA distribution

ggplot(VF_behav_patientonly, aes(x = ASIA_score)) +

  xlab("ASIA impairment scale") +

  ylab("Number of participants") +

  geom_bar(fill = "#F59884", color = "black", width = 1) +  # Add border with 'color'

  theme_minimal() +

  scale_y_continuous(breaks = scales::pretty_breaks(n = 10),

                     expand = c(0, 0)) +

  theme(

    axis.title = element_text(size=17),

    axis.text = element_text(size = 17)

  )



# Behavioral comparisons --------------------------------------------------

# Pathological VF percent rank according to Regensburger Wortflüssigkeitstest (RWT) norm values ---------------

#Calculate the mean percentile ranks

VF_behav$MeanPR <- rowMeans(VF_behav[, c("PR_B", "PR_M", "PR_K", "PR_P", "PR_S")])


#Boxplot with age- and education adjusted percentile ranks

ggplot(VF_behav, aes(x = Group, y = MeanPR)) +

  geom_boxplot(outlier.shape=NA) +

  labs(x = "Group", y = "Verbal fluency performance \n mean percentile rank [%]") +

  geom_jitter(data = VF_behav, aes(x = Group, y = MeanPR),    # Add single data points (jitter for better visibility)

              width = 0.15, height =0, size = 2, color = "black", alpha = 0.6) +

  scale_x_discrete(labels = c("Non-SCI" = "Non-injured \n controls", "SCI" = "Spinal cord injury")) +  # <-- new labels

  theme_minimal() +

  theme(plot.title = element_text(hjust = 0.5),

        axis.title = element_text(size = 21),

        axis.text = element_text(size = 21))


#Number of pathological percentile ranks (PR<=10, according to Lezak et al.) per person out of five letters

VF_behav$count_PR_smallerequal_10 <- rowSums(VF_behav[, c("PR_B", "PR_M", "PR_K","PR_P","PR_S")] <= 10)

aggregate(count_PR_smallerequal_10 ~ Group, data = VF_behav, FUN = mean)


leveneTest(VF_behav$count_PR_smallerequal_10, VF_behav$Group)  #homogeneity of variances is fulfilled

by(VF_behav$count_PR_smallerequal_10,VF_behav$Group, shapiro.test) #not fulfilled


wilcox.test(count_PR_smallerequal_10~Group,data=VF_behav)


#Boxplot of pathological percentile ranks per group

ggplot(VF_behav, aes(x = Group, y = count_PR_smallerequal_10)) +
```

```r
  geom_boxplot(outlier.shape=NA) +
  geom_signif(comparisons = list(c("Non-SCI", "SCI")),
              annotations = "p=0.021",
              y_position = 5, textsize = 6) +
  labs(x = "Group", y = "Number of below-average\n performances out of 5 letters") +
  geom_jitter(data = VF_behav, aes(x = Group, y = count_PR_smallerequal_10),   # Add single data points (jitter for better vi
sibility)
               width = 0.15, height =0, size = 2, color = "black", alpha = 0.6) +
  scale_x_discrete(labels = c("Non-SCI" = "Non-injured \n controls", "SCI" = "Spinal cord injury")) +  # <-- new labels
  theme_minimal() +
  theme(plot.title = element_text(hjust = 0.5),
        axis.title = element_text(size = 21),
        axis.text = element_text(size = 21))



# Compare sum of correct words --------------------------------------------
#Check assumptions for t-test
leveneTest(VF_behav$Sum_Correctwords, VF_behav$Group)


by(VF_behav$Sum_Correctwords, VF_behav$Group, shapiro.test)


wilcox.test(Sum_Correctwords~Group,data=VF_behav)


#Boxplot showing sum of correct words per group
ggplot(VF_behav, aes(x = Group, y = Sum_Correctwords)) +
  geom_boxplot(outlier.shape=NA) +  #remove outlier datapoint, when plotting all single data points
  geom_signif(comparisons = list(c("Non-SCI", "SCI")),
              annotations = "p=0.024",
              y_position = 185, textsize = 6) +
  geom_jitter(data = VF_behav, aes(x = Group, y = Sum_Correctwords),
              width = 0.15, height =0, size = 2, color = "black", alpha = 0.6) +
  labs(x = "Group", y = "Sum of correct words") +
  theme_minimal() +
  scale_x_discrete(labels = c("Non-SCI" = "Non-injured \n controls", "SCI" = "Spinal cord injury")) +
  theme(plot.title = element_text(hjust = 0.5),
        axis.title = element_text(size = 21),
        axis.text = element_text(size = 21))



# Beta weights and functional connectivity values extracted from SPM and CONN -----------------------------------
#Read beta weights from SPM second level analysis Task condition
VF_behav_withoutsubject7 <-VF_behav[VF_behav$ID != 'SCI-Cog_07' ,] #remove these two subjects as they were extreme outliers i
n the imaging data
VF_behav_withoutsubject7and33 <-VF_behav_withoutsubject7[VF_behav_withoutsubject7$ID != 'SCI-Cog_33' ,]


#Beta weights excluding subject 7 and 33
spm_mat_rightputamen <- readMat("D:/Main_study/VF_SPM/Second_level_Task_SCI-HC_withoutsubject33and7/rightputamen_betaweights.
mat")



ID_SCI <- VF_behav_withoutsubject7and33 %>% filter(Group == "SCI") %>% pull(ID)
ID_nonSCI <- VF_behav_withoutsubject7and33 %>% filter(Group == "Non-SCI") %>% pull(ID)
```

```
SPM_subject_IDs <- c(ID_SCI,ID_nonSCI) #get the order of the IDs specified in the SPM contrast


beta_weight_dataframe<- data.frame(

                                    Beta_weights_rightputamen=as.vector(spm_mat_rightputamen$Y),

                                    ID=SPM_subject_IDs)


VF_behav_withoutsubject7and33 <- VF_behav_withoutsubject7and33 %>%  #add the IDs

  left_join(beta_weight_dataframe, by = "ID")


beta_weight_dataframe <- beta_weight_dataframe %>%

  left_join(VF_behav_withoutsubject7and33 %>% dplyr::select(ID, Group, Age), by = "ID")


#Functional connectivity between cingulate gyrus and right insula in SCI individuals not taking pain medication (for correlat
ion with verbal fluency performance)

VF_behav$FC_rIns_cingulategyrus_SCInopainmed <- c(NaN, NaN, NaN, NaN, NaN, NaN, NaN, NaN, NaN, NaN, 0.0726925, 0.189839, NaN,
NaN, NaN, NaN, 0.338328, NaN, NaN,0.224544, 0.325745, 0.22154, NaN, NaN, 0.13007, NaN, 0.191054, 0.235024, NaN, NaN, NaN, NaN
, NaN, NaN, NaN, NaN, NaN, NaN, NaN, NaN,0.0805181, NaN, NaN, 0.65131, NaN, 0.185235, 0.230586, NaN, 0.252576, NaN, NaN, NaN)

#Extracted from the second level covariates in CONN setup (FC calculated as group difference after removing subject 7,33 and
controlling for age)

VF_behav_withoutsubject7and33$FC_linsula_lSFG <- c(0.158235, -0.0689144, 0.0177375, -0.0151064, 0.345695, 0.188939, 0.176241,
0.0492458, 0.0745281, -0.0474487, 0.0103991, 0.107647, 0.329286, 0.00836637, 0.231383, -0.112062, 0.160815, 0.0357983, 0.0837
409, 0.00266352, 0.227808, 0.196094, 0.385138, -0.149254, 0.104473, -0.0752449, -0.0665756, 0.320181, 0.162207, 0.048383, 0.2
76075, 0.0551514, 0.166329, 0.134789, 0.11675, 0.111751, 0.259815, 0.0941061, 0.0113287, 0.191102, 0.137308, -0.0812785, 0.38
1544, 0.11795, -0.045338, 0.100152, 0.119419, 0.230444, 0.139406, 0.0206131)

VF_behav_withoutsubject7and33$FC_lpostCG_lputamen_corr_roitoroi <- c(NaN, -0.0267439, 0.03487, NaN, NaN, NaN, NaN, NaN, 0.115
167, 0.201466, 0.152538, 0.444588, NaN, -0.00329638, NaN, 0.0028795, NaN, NaN, 0.125531, 0.273472, 0.171991, NaN, NaN, 0.0790
756, NaN, 0.17655, 0.115803, NaN, NaN, -0.129628, 0.216883, 0.15612, NaN, NaN, NaN, NaN, NaN, 0.286326, 0.00887874, NaN, NaN,
0.522477, NaN, 0.15981, 0.324858, NaN, 0.0651534, NaN, NaN, 0.0669894)



#FC values in SCI for correlation with verbal fluency performance

VF_behav_withoutsubject7and33$FC_SCI_rInsula_postCG <- c(NaN, -0.0849023, 0.0954357, NaN, NaN, NaN, NaN, NaN, 0.204989, -0.13
13, 0.122166, 0.362844, NaN, -0.00699866, NaN, 0.114557, NaN, NaN, 0.0947221, 0.308536, -0.123677, NaN, NaN, 0.114861, NaN, 0
.190167, 0.176769, NaN, NaN, 0.0221591, 0.0532834, 0.326007, NaN, NaN, NaN, NaN, NaN, 0.243089, 0.041787, NaN, NaN, 0.634194,
NaN, 0.168984, 0.389035, NaN, 0.170234, NaN, NaN, 0.124092)

VF_behav_withoutsubject7and33$FC_SCI_precuneous_AC <- c(NaN, 0.243575, 0.107796, NaN, NaN, NaN, NaN, NaN, 0.340626, -0.081640
3, 0.0419649, 0.249929, NaN, 0.135932, NaN, 0.0884027, NaN, NaN,0.298426, 0.431004, 0.0149112, NaN, NaN, 0.0519302, NaN, 0.34
7318, 0.0615563, NaN, NaN, -0.119798, 0.0260801, 0.306845, NaN, NaN, NaN, NaN, NaN, 0.197023, -0.0220959, NaN, NaN, 0.443416,
NaN, -0.218137, 0.154544, NaN, -0.0312167, NaN, NaN, 0.243369)

VF_behav_withoutsubject7and33$FC_SCI_lputamen_rPreCG <- c(NaN, 0.0164896, 0.0154655, NaN, NaN, NaN, NaN, NaN, 0.0695568, -0.0
483085, 0.19858, 0.388738, NaN, 0.0421072, NaN, 0.19111, NaN, NaN, 0.182595, 0.275245, 0.0779917, NaN, NaN, 0.0484757, NaN, 0
.209681, 0.0827674, NaN, NaN, 0.145473, -0.0341182, 0.254366, NaN, NaN, NaN, NaN, NaN, 0.399489, -0.0416616, NaN, NaN, 0.5420
1, NaN, 0.0913235, 0.295248, NaN, 0.00373442, NaN, NaN, 0.174418)

VF_behav_withoutsubject7and33$FC_SCI_lputamen_lPreCG <- c(NaN, -0.125252, -0.00989556, NaN, NaN, NaN, NaN, NaN, 0.0828187, -0
.0270373, 0.235828, 0.194382, NaN, -0.0570909, NaN, 0.0334785, NaN, NaN, 0.147339, 0.207008, 0.0508029, NaN, NaN, -0.020522,
NaN, 0.204708, 0.0747552, NaN, NaN, -0.186079, 0.121641, 0.368445, NaN, NaN, NaN, NaN, NaN, 0.383605, -0.0802677, NaN, NaN, 0
.47743, NaN, 0.00292649, 0.170852, NaN, 0.225178, NaN, NaN, 0.176656)



VF_behav_withoutsubject7and33_patientonly <- VF_behav_withoutsubject7and33[VF_behav_withoutsubject7and33$Group=="SCI",]

VF_behav_withoutsubject7and33_controlsonly <- VF_behav_withoutsubject7and33[VF_behav_withoutsubject7and33$Group=="Non-SCI",]


# Correlate beta weights with verbal fluency performance ---------------------------------

#Check assumptions



shapiro.test(VF_behav_withoutsubject7and33$Beta_weights_rightputamen) #normal

shapiro.test(VF_behav_withoutsubject7and33_patientonly$Beta_weights_rightputamen) #normal

shapiro.test(VF_behav_withoutsubject7and33_controlsonly$Beta_weights_rightputamen) #normal


shapiro.test(VF_behav_withoutsubject7and33$Sum_Correctwords) #normal

shapiro.test(VF_behav_withoutsubject7and33_patientonly$Sum_Correctwords) #normal

shapiro.test(VF_behav_withoutsubject7and33_controlsonly$Sum_Correctwords) #non-normal
```

```
partialcor <- pcor.test(VF_behav_withoutsubject7and33$Sum_Correctwords, VF_behav_withoutsubject7and33[,"Beta_weights_rightput
amen"], VF_behav_withoutsubject7and33[,"Age"], method = "pearson")


ggplot(VF_behav_withoutsubject7and33, aes(x = Beta_weights_rightputamen, y = Sum_Correctwords, color = Group)) +

  geom_point() +

  geom_smooth(method = "lm", se = FALSE, color = "black") +  # Add regression line

  theme_minimal()+

  theme(

    axis.text.x = element_text(size = 14, angle = 0, vjust = 0.5, hjust = 1),

    axis.text.y = element_text(size = 14),

    axis.title.x = element_text(size = 16),

    axis.title.y = element_text(size = 16),

    legend.text = element_text(size = 14),

    legend.title = element_text(size = 16),

    legend.position = c(0.75, 0.13),  # Move legend to upper-left corner

    legend.background = element_rect(fill = "white", color = "black")

  ) +

  scale_color_discrete(labels = c("Non-SCI" = "Non-injured controls", "SCI" = "Spinal cord injury"))+

  labs(x = "Beta weights of significant cluster", y = "Sum of correct words")# +



# Correlate functional connectivity with verbal fluency performance -------------------------------------------

shapiro.test(VF_behav_withoutsubject7and33$FC_linsula_lSFG) #normal

shapiro.test(VF_behav_withoutsubject7and33_patientonly$FC_linsula_lSFG) #normal

shapiro.test(VF_behav_withoutsubject7and33_controlsonly$FC_linsula_lSFG) #normal

shapiro.test(VF_behav_withoutsubject7and33_patientonly$FC_lpostCG_lputamen_corr_roitoroi) #normal


ggplot(VF_behav_withoutsubject7and33_patientonly, aes(x = FC_SCI_rInsula_postCG, y = Sum_Correctwords)) +

  geom_point() +

  geom_smooth(method = "lm", se = FALSE, color = "black") +  # Add regression line

  theme_minimal()+

  theme(

    axis.text.x = element_text(size = 16, angle = 0, vjust = 0.5, hjust = 1),

    axis.text.y = element_text(size = 16),

    axis.title.x = element_text(size = 16),

    axis.title.y = element_text(size = 16)

  ) +

  labs( x = "FC between right insula and right PoCG", y = "Sum of correct words")+

  annotate("text", x = Inf, y = Inf, label = "",

           hjust = 1.1, vjust = 1.1, size = 4, color = "black")


ggplot(VF_behav_withoutsubject7and33_patientonly, aes(x = FC_SCI_precuneous_AC, y = Sum_Correctwords)) +

  geom_point() +

  geom_smooth(method = "lm", se = FALSE, color = "black") +  # Add regression line

  theme_minimal()+

  theme(

    axis.text.x = element_text(size = 16, angle = 0, vjust = 0.5, hjust = 1),

    axis.text.y = element_text(size = 16),

    axis.title.x = element_text(size = 16),

    axis.title.y = element_text(size = 16)

  ) +
```

```r
  labs( x = "FC between precuneus and ACG", y = "Sum of correct words")+

  annotate("text", x = Inf, y = Inf, label = "",

           hjust = 1.1, vjust = 1.1, size = 4, color = "black")


ggplot(VF_behav_withoutsubject7and33_patientonly, aes(x = FC_SCI_lputamen_rPreCG, y = Sum_Correctwords)) +

  geom_point() +

  geom_smooth(method = "lm", se = FALSE, color = "black") +  # Add regression line

  theme_minimal()+

  theme(

    axis.text.x = element_text(size = 16, angle = 0, vjust = 0.5, hjust = 1),

    axis.text.y = element_text(size = 16),

    axis.title.x = element_text(size = 16),

    axis.title.y = element_text(size = 16)

  ) +

  labs(x = "FC between left putamen and right PreCG", y = "Sum of correct words")+

  annotate("text", x = Inf, y = Inf, label = "",

           hjust = 1.1, vjust = 1.1, size = 4, color = "black")


ggplot(VF_behav_withoutsubject7and33_patientonly, aes(x = FC_SCI_lputamen_lPreCG, y = Sum_Correctwords)) +

  geom_point() +

  geom_smooth(method = "lm", se = FALSE, color = "black") +  # Add regression line

  theme_minimal()+

  theme(

    axis.text.x = element_text(size = 16, angle = 0, vjust = 0.5, hjust = 1),

    axis.text.y = element_text(size = 16),

    axis.title.x = element_text(size = 16),

    axis.title.y = element_text(size = 16)

  ) +

  labs(x = "FC between left putamen and left PreCG", y = "Sum of correct words")+

  annotate("text", x = Inf, y = Inf, label = "",

           hjust = 1.1, vjust = 1.1, size = 4, color = "black")




cor.test(VF_behav_withoutsubject7and33_patientonly$Sum_Correctwords,VF_behav_withoutsubject7and33_patientonly[,"FC_SCI_rInsula_postCG"], method = 'pearson')

cor.test(VF_behav_withoutsubject7and33_patientonly$Sum_Correctwords,VF_behav_withoutsubject7and33_patientonly[,"FC_SCI_precuneous_AC"], method = 'pearson')

cor.test(VF_behav_withoutsubject7and33_patientonly$Sum_Correctwords,VF_behav_withoutsubject7and33_patientonly[,"FC_SCI_lputamen_rPreCG"], method = 'pearson')

cor.test(VF_behav_withoutsubject7and33_patientonly$Sum_Correctwords,VF_behav_withoutsubject7and33_patientonly[,"FC_SCI_lputamen_lPreCG"], method = 'pearson')

cor.test(VF_behav_withoutsubject7and33_patientonly$Sum_Correctwords,VF_behav_withoutsubject7and33_patientonly[,"FC_lpostCG_lputamen_corr_roitoroi"], method = 'pearson')




# Subgroup analysis: Without lumbar SCI -----------------------------------

VF_behav_withoutsubject2 <-VF_behav[VF_behav$ID != 'SCI-Cog_02' ,] #remove person with lumbar SCI


#Compare Sum of correct words

#Check assumptions for t-test

leveneTest(VF_behav_withoutsubject2$Sum_Correctwords, VF_behav_withoutsubject2$Group)


# Normality assumption: Non-SCI group non-normally distributed
```

```r
by(VF_behav_withoutsubject2$Sum_Correctwords, VF_behav_withoutsubject2$Group, shapiro.test) #non-normal

wilcox.test(Sum_Correctwords~Group,data=VF_behav_withoutsubject2)

ggplot(VF_behav_withoutsubject2, aes(x = Group, y = Sum_Correctwords)) +
  geom_boxplot(outlier.shape=NA) +
  geom_signif(comparisons = list(c("Non-SCI", "SCI")),
              annotations = "p=0.019",
              y_position = 185, textsize = 5) +
  geom_jitter(data = VF_behav_withoutsubject2, aes(x = Group, y = Sum_Correctwords),
              width = 0.15, height =0, size = 2, color = "black", alpha = 0.6) +
  labs(x = "Group", y = "Sum of correct words") +
  theme_minimal() +
  scale_x_discrete(labels = c("Non-SCI" = "Non-injured controls", "SCI" = "Spinal cord injury")) +
  theme(plot.title = element_text(hjust = 0.5),
        axis.title = element_text(size = 17),
        axis.text = element_text(size = 15))



#Normative data of RWT: without subject 2 (lesion L1)
leveneTest(VF_behav_withoutsubject2$count_PR_smallerequal_10, VF_behav_withoutsubject2$Group)  #homogeneity of variances is f
ulfilled
by(VF_behav_withoutsubject2$count_PR_smallerequal_10,VF_behav_withoutsubject2$Group, shapiro.test) #not fulfilled

wilcox.test(count_PR_smallerequal_10~Group,data=VF_behav_withoutsubject2)

ggplot(VF_behav_withoutsubject2, aes(x = Group, y = count_PR_smallerequal_10)) +
  geom_boxplot(outlier.shape=NA) +
  geom_signif(comparisons = list(c("Non-SCI", "SCI")),
              annotations = "p=0.022",
              y_position = 5, textsize = 5) +
  labs(x = "Group", y = "Number of below-average\n performances out of 5 letters") +
  scale_x_discrete(labels = c("Non-SCI" = "Non-injured controls", "SCI" = "Spinal cord injury")) +  # <-- new labels
  geom_jitter(data = VF_behav_withoutsubject2, aes(x = Group, y = count_PR_smallerequal_10),
              width = 0.15, height =0, size = 2, color = "black", alpha = 0.6) +
  theme_minimal() +
  theme(plot.title = element_text(hjust = 0.5),
        axis.title = element_text(size = 17),
        axis.text = element_text(size = 15))



# Subgroup analysis ASIA A and D -----------------------------------------
#Number of pathological percentile ranks (PR<=10, according to Lezak et al.) per person out of five letters
VF_behav_patientonly$count_PR_smallerequal_10 <- rowSums(VF_behav_patientonly[, c("PR_B", "PR_M", "PR_K","PR_P","PR_S")] <= 1
0)

ASIA_results <- ASIA_results[ ASIA_results$ID %in% VF_behav_patientonly$ID  , ] #only filter for patients
VF_behav_patient_ASIA <- merge(VF_behav_patientonly,ASIA_results,by="ID")
VF_behav_patient_ASIA$ASIA <- as.factor(VF_behav_patient_ASIA$ASIA)

VF_behav_patientonly$ASIA_score <- droplevels(VF_behav_patientonly$ASIA_score)
```

```r
VF_behav_patient_ASIA_A_D <- VF_behav_patient_ASIA[VF_behav_patient_ASIA$ASIA == 'A' | VF_behav_patient_ASIA$ASIA== 'D',]

VF_behav_patient_ASIA_A_D$ASIA <- droplevels(VF_behav_patient_ASIA_A_D$ASIA)


VF_behav_patient_ASIA <- VF_behav_patient_ASIA %>%  #Convert scores into numeric

  mutate_at(vars(Touch_right,Touch_left,Touch_total,Pain_left,Pain_right,Pain_total,Motor_right,Motor_left,Motor_total), as.n
umeric)


# Demographic and clinical characteristics

mean(VF_behav_patient_ASIA_A_D$Age[VF_behav_patient_ASIA_A_D$ASIA=="A"])

mean(VF_behav_patient_ASIA_A_D$Age[VF_behav_patient_ASIA_A_D$ASIA=="D"])

sd(VF_behav_patient_ASIA_A_D$Age[VF_behav_patient_ASIA_A_D$ASIA=="A"])

sd(VF_behav_patient_ASIA_A_D$Age[VF_behav_patient_ASIA_A_D$ASIA=="D"])

mean(VF_behav_patient_ASIA_A_D$TSI[VF_behav_patient_ASIA_A_D$ASIA=="A"])

sd(VF_behav_patient_ASIA_A_D$TSI[VF_behav_patient_ASIA_A_D$ASIA=="A"])

mean(VF_behav_patient_ASIA_A_D$TSI[VF_behav_patient_ASIA_A_D$ASIA=="D"])

sd(VF_behav_patient_ASIA_A_D$TSI[VF_behav_patient_ASIA_A_D$ASIA=="D"])


#Test for difference of age between the two groups

wilcox_test(Age~ASIA,data=VF_behav_patient_ASIA_A_D)


#Test for difference of age between the two groups

wilcox_test(TSI~ASIA,data=VF_behav_patient_ASIA_A_D)


#Test for difference of Sex between the two groups

table(VF_behav_patient_ASIA_A_D$ASIA, VF_behav_patient_ASIA_A_D$Sex)

chisq.test(VF_behav_patient_ASIA_A_D$Sex,VF_behav_patient_ASIA_A_D$ASIA,correct=FALSE)


#Compare lesion levels

contingency_table <- data.frame("A"=c(0,2,9),"D"=c(4,6,2),row.names=c("cervival1_4","cervical5_8","thoracic"))

mosaicplot(contingency_table,color=TRUE)

chisq.test(contingency_table)


# Calculate Chi square test if there are significant differences in education level between the groups

aggregate(VF_behav_patient_ASIA_A_D$Education,by=list(VF_behav_patient_ASIA_A_D$ASIA,VF_behav_patient_ASIA_A_D$Education),FUN
=length)

contingency_table <- data.frame("A"=c(1,3,2,2,3),"D"=c(0,5,2,1,4),row.names=c("Obligatorische_Schule","Berufslehre","Mittelsc
hule","Hoehere_Berufsbildung","Universitaet"))

mosaicplot(contingency_table,color=TRUE)

chisq.test(contingency_table)


table(VF_behav_patient_ASIA_A_D$ASIA)

wilcox_test(Sum_Correctwords~ASIA,data=VF_behav_patient_ASIA_A_D) #p=0.46


#Boxplot showing sum of correct words between ASIA A and D

ggplot(VF_behav_patient_ASIA_A_D, aes(x = ASIA, y = Sum_Correctwords)) +

  geom_boxplot(outlier.shape=NA) +

  geom_signif(comparisons = list(c("A", "D")),

              annotations = "p=0.46",

              y_position = 140, textsize = 5) +

  labs(x = "AIS level", y = "Sum of correct words") +

  geom_jitter(data = VF_behav_patient_ASIA_A_D, aes(x = ASIA, y = Sum_Correctwords),

              width = 0.15, height =0, size = 2, color = "black", alpha = 0.6) +
```

```
  theme_minimal() +
  theme(plot.title = element_text(hjust = 0.5),
        axis.title = element_text(size = 17),
        axis.text = element_text(size = 15))



#Compare normative data
leveneTest(VF_behav_patient_ASIA_A_D$count_PR_smallerequal_10, VF_behav_patient_ASIA_A_D$ASIA)  #homogeneity of variances is fulfilled
by(VF_behav_patient_ASIA_A_D$count_PR_smallerequal_10,VF_behav_patient_ASIA_A_D$ASIA, shapiro.test) #not fulfilled


wilcox.test(count_PR_smallerequal_10~ASIA,data=VF_behav_patient_ASIA_A_D)  #p=0.838


ggplot(VF_behav_patient_ASIA_A_D, aes(x = ASIA, y = count_PR_smallerequal_10)) +
  geom_boxplot(outlier.shape=NA)+
  labs(x = "AIS level", y = "Number of below-average\n performances out of 5 letters") +
  geom_jitter(data = VF_behav_patient_ASIA_A_D, aes(x = ASIA, y = count_PR_smallerequal_10),
              width = 0.15, height =0, size = 2, color = "black", alpha = 0.6) +
  theme_minimal() +
  theme(plot.title = element_text(hjust = 0.5),
        axis.title = element_text(size = 17),
        axis.text = element_text(size = 15))+
  geom_signif(comparisons = list(c("A", "D")),
              annotations = "p=0.838",  #adapt p-value
              y_position = 5, textsize = 5)



VF_behav_patient_ASIA_A_D <- VF_behav_patient_ASIA_A_D %>%  #Convert scores into numeric
  mutate_at(vars(Touch_right,Touch_left,Touch_total,Pain_left,Pain_right,Pain_total,Motor_right,Motor_left,Motor_total), as.numeric)


#ISNCSCI scores
summary_stats_AIS_A <- VF_behav_patient_ASIA %>%
  filter(ASIA == "A") %>%
  summarise(
    mean_beruehrung = mean(Touch_total, na.rm = TRUE),
    sd_beruehrung = sd(Touch_total, na.rm = TRUE),
    mean_pinprick = mean(Pain_total, na.rm = TRUE),
    sd_pinprick= sd(Pain_total, na.rm = TRUE),
    mean_motorik = mean(Motor_total, na.rm = TRUE),
    sd_motorik = sd(Motor_total, na.rm = TRUE)
  )


summary_stats_AIS_A


summary_stats_AIS_D <- VF_behav_patient_ASIA %>%
  filter(ASIA == "D") %>%
  summarise(
    mean_beruehrung = mean(Touch_total, na.rm = TRUE),
    sd_beruehrung = sd(Touch_total, na.rm = TRUE),
    mean_pinprick = mean(Pain_total, na.rm = TRUE),
```

```
    sd_pinprick= sd(Pain_total, na.rm = TRUE),

    mean_motorik = mean(Motor_total, na.rm = TRUE),

    sd_motorik = sd(Motor_total, na.rm = TRUE)

  )


summary_stats_AIS_D




wilcox.test(Touch_total~ASIA,data=VF_behav_patient_ASIA_A_D)

wilcox.test(Pain_total~ASIA,data=VF_behav_patient_ASIA_A_D)

wilcox.test(Motor_total~ASIA,data=VF_behav_patient_ASIA_A_D)




#Difference in medication intake

# Calculate Chi square test if there are significant differences in medication

bladder_med_table <- matrix(c(5,6,1,11),nrow=2,byrow=TRUE) #order SCI took med, SCI did not take med, HC took med, HC did not take med

chisq.test(bladder_med_table)


antimuscarinics_med_table <- matrix(c(5,6,4,8),nrow=2,byrow=TRUE) #order SCI took med, SCI did not take med, HC took med, HC did not take med

chisq.test(antimuscarinics_med_table)


pain_med_table <- matrix(c(5,6,4,8),nrow=2,byrow=TRUE) #order SCI took med, SCI did not take med, HC took med, HC did not take med

chisq.test(pain_med_table)


bloodpressure_med_table <- matrix(c(1,10,4,8),nrow=2,byrow=TRUE) #order SCI took med, SCI did not take med, HC took med, HC did not take med

chisq.test(bloodpressure_med_table)


spasticity_med_table <- matrix(c(1,10,3,9),nrow=2,byrow=TRUE) #order SCI took med, SCI did not take med, HC took med, HC did not take med

chisq.test(spasticity_med_table)


constipation_med_table <- matrix(c(1,10,1,11),nrow=2,byrow=TRUE) #order SCI took med, SCI did not take med, HC took med, HC did not take med

chisq.test(constipation_med_table)


others_med_table <- matrix(c(2,9,3,9),nrow=2,byrow=TRUE) #order SCI took med, SCI did not take med, HC took med, HC did not take med

chisq.test(others_med_table)




# Subgroup analysis individuals with vs without pain medication -----------

table(VF_behav_patientonly$Pain_medication)


#Demographic and clinical characteristics

#Test for difference of Sex between the two groups

table(VF_behav_patientonly$Pain_medication, VF_behav_patientonly$Sex)

chisq.test(VF_behav_patientonly$Sex,VF_behav_patientonly$Pain_medication,correct=FALSE)


#Age distribution

mean(VF_behav_patientonly$Age[VF_behav_patientonly$Pain_medication=="0"])
```

```
sd(VF_behav_patientonly$Age[VF_behav_patientonly$Pain_medication=="0"])

mean(VF_behav_patientonly$Age[VF_behav_patientonly$Pain_medication=="1"])

sd(VF_behav_patientonly$Age[VF_behav_patientonly$Pain_medication=="1"])


#Test for difference of age between the two groups

wilcox_test(Age~Pain_medication,data=VF_behav_patientonly)


#TSI distribution

mean(VF_behav_patientonly$TSI[VF_behav_patientonly$Pain_medication=="0"])

sd(VF_behav_patientonly$TSI[VF_behav_patientonly$Pain_medication=="0"])


mean(VF_behav_patientonly$TSI[VF_behav_patientonly$Pain_medication=="1"])

sd(VF_behav_patientonly$TSI[VF_behav_patientonly$Pain_medication=="1"])


#Test for difference of TSI between the two groups

wilcox_test(TSI~Pain_medication,data=VF_behav_patientonly)


#Compare pain level

mean(VF_behav_patientonly$pain[VF_behav_patientonly$Pain_medication=="1"])

sd(VF_behav_patientonly$pain[VF_behav_patientonly$Pain_medication=="1"])


mean(VF_behav_patientonly$pain[VF_behav_patientonly$Pain_medication=="0"])

sd(VF_behav_patientonly$pain[VF_behav_patientonly$Pain_medication=="0"])


t.test(pain~Pain_medication,data=VF_behav_patientonly)


#Compare STAI trait levels

mean(VF_behav_patientonly$sum_stai_trait[VF_behav_patientonly$Pain_medication=="1"])

sd(VF_behav_patientonly$sum_stai_trait[VF_behav_patientonly$Pain_medication=="1"])


mean(VF_behav_patientonly$sum_stai_trait[VF_behav_patientonly$Pain_medication=="0"])

sd(VF_behav_patientonly$sum_stai_trait[VF_behav_patientonly$Pain_medication=="0"])


t.test(sum_stai_trait~Pain_medication,data=VF_behav_patientonly)


#Compare STAI state levels

mean(VF_behav_patientonly$sum_stai_state[VF_behav_patientonly$Pain_medication=="1"])

sd(VF_behav_patientonly$sum_stai_state[VF_behav_patientonly$Pain_medication=="1"])


mean(VF_behav_patientonly$sum_stai_state[VF_behav_patientonly$Pain_medication=="0"])

sd(VF_behav_patientonly$sum_stai_state[VF_behav_patientonly$Pain_medication=="0"])


t.test(sum_stai_state~Pain_medication,data=VF_behav_patientonly)


# Calculate Chi square test if there are significant differences in education level between the groups

aggregate(VF_behav_patientonly$Education,by=list(VF_behav_patientonly$Pain_medication,VF_behav_patientonly$Education),FUN=len
gth)

contingency_table <- data.frame("0"=c(1,5,2,4,3),"1"=c(0,4,2,1,4),row.names=c("Obligatorische_Schule","Berufslehre","Mittelsc
hule","Hoehere_Berufsbildung","Universitaet"))

mosaicplot(contingency_table,color=TRUE)

chisq.test(contingency_table) #no difference
```

```r
#ISNCSCI scores
summary_stats_INSCSCI_painmed_0 <- VF_behav_patient_ASIA %>%
  filter(Pain_medication == "0") %>%
  summarise(
    mean_beruehrung = mean(Touch_total, na.rm = TRUE),
    sd_beruehrung = sd(Touch_total, na.rm = TRUE),
    mean_pinprick = mean(Pain_total, na.rm = TRUE),
    sd_pinprick= sd(Pain_total, na.rm = TRUE),
    mean_motorik = mean(Motor_total, na.rm = TRUE),
    sd_motorik = sd(Motor_total, na.rm = TRUE)
  )

summary_stats_INSCSCI_painmed_0

summary_stats_INSCSCI_painmed_1 <- VF_behav_patient_ASIA %>%
  filter(Pain_medication == "1") %>%
  summarise(
    mean_beruehrung = mean(Touch_total, na.rm = TRUE),
    sd_beruehrung = sd(Touch_total, na.rm = TRUE),
    mean_pinprick = mean(Pain_total, na.rm = TRUE),
    sd_pinprick= sd(Pain_total, na.rm = TRUE),
    mean_motorik = mean(Motor_total, na.rm = TRUE),
    sd_motorik = sd(Motor_total, na.rm = TRUE)
  )

summary_stats_INSCSCI_painmed_1

wilcox.test(Touch_total~Pain_medication,data=VF_behav_patient_ASIA)
wilcox.test(Pain_total~Pain_medication,data=VF_behav_patient_ASIA)
wilcox.test(Motor_total~Pain_medication,data=VF_behav_patient_ASIA)

#Check assumptions for t-test
leveneTest(VF_behav_patientonly$Sum_Correctwords, VF_behav_patientonly$Pain_medication)

# Normality assumption
by(VF_behav_patientonly$Sum_Correctwords, VF_behav_patientonly$Pain_medication, shapiro.test) #normal

t.test(Sum_Correctwords~Pain_medication,data=VF_behav_patientonly)

ggplot(VF_behav_patientonly, aes(x = Pain_medication, y = Sum_Correctwords)) +
  geom_boxplot(outlier.shape=NA) +
  labs(x = "SCI groups", y = "Sum of correct words") +
  theme_minimal() +
  geom_signif(comparisons = list(c("0", "1")),
              annotations = "p=0.523",  #adapt p-value
              y_position = 140, textsize = 5) +
  geom_jitter(data = VF_behav_patientonly, aes(x = Pain_medication, y = Sum_Correctwords),
              width = 0.15, height =0, size = 2, color = "black", alpha = 0.6) +
```

```
  scale_x_discrete(labels = c("0" = "Without pain\n medication", "1" = "With pain medication")) +
  theme(plot.title = element_text(hjust = 0.5),
        axis.title = element_text(size = 17),
        axis.text = element_text(size = 15))

#Pathological comparison SCI with vs without pain medication
leveneTest(VF_behav_patientonly$count_PR_smallerequal_10, VF_behav_patientonly$Pain_medication)  #homogeneity of variances is fulfilled
by(VF_behav_patientonly$count_PR_smallerequal_10,VF_behav_patientonly$Pain_medication, shapiro.test) #not fulfilled

wilcox.test(count_PR_smallerequal_10~Pain_medication,data=VF_behav_patientonly) #p=0.537

#Boxplot
ggplot(VF_behav_patientonly, aes(x = Pain_medication, y = count_PR_smallerequal_10)) +
  geom_boxplot(outlier.shape=NA) +
  labs(x = "SCI groups", y = "Number of below-average\n performances out of 5 letters") +
  scale_x_discrete(labels = c("0" = "Without pain\n medication", "1" = "With pain medication")) +  # <-- new labels
  geom_jitter(data = VF_behav_patientonly, aes(x = Pain_medication, y = count_PR_smallerequal_10),
              width = 0.15, height =0, size = 2, color = "black", alpha = 0.6) +
  theme_minimal() +
  geom_signif(comparisons = list(c("0", "1")),
              annotations = "p=0.537",  #adapt p-value
              y_position = 5, textsize = 5) +
  theme(plot.title = element_text(hjust = 0.5),
        axis.title = element_text(size = 17),
        axis.text = element_text(size = 15))

#Correlation of FC with VF performance (only significant in SCI without pain medication and right insula (seed) with cingulate gyrus)
VF_behav_patientonly_FC <- VF_behav[VF_behav$Group == 'SCI',]
VF_behav_SCI_nopainmed <- VF_behav_patientonly_FC[VF_behav_patientonly_FC$Pain_medication=="0",]

shapiro.test(VF_behav_SCI_nopainmed$Sum_Correctwords) #normal
shapiro.test(VF_behav_SCI_nopainmed$FC_rIns_cingulategyrus_SCInopainmed) #non-normal

cor.test(VF_behav_SCI_nopainmed$Sum_Correctwords,VF_behav_SCI_nopainmed[,"FC_rIns_cingulategyrus_SCInopainmed"], method = 'spearman')

ggplot(VF_behav_SCI_nopainmed, aes(x = FC_rIns_cingulategyrus_SCInopainmed, y = Sum_Correctwords)) +
  geom_point() +
  geom_smooth(method = "lm", se = FALSE, color = "black") +  # Add regression line
  theme_minimal()+
  theme(
    axis.text.x = element_text(size = 14, angle = 0, vjust = 0.5, hjust = 1),
    axis.text.y = element_text(size = 14),
    axis.title.x = element_text(size = 16),
    axis.title.y = element_text(size = 16),
    legend.text = element_text(size = 14),
    legend.title = element_text(size = 16),
    legend.position = c(0.75, 0.13),
    legend.background = element_rect(fill = "white", color = "black")
  ) +  labs(x = "FC between right Insula and Cingulate gyrus", y = "Sum of correct words")
```

```r
# Subgroup analysis: Cervical vs thoracic ---------------------------------

VF_behav <- VF_behav %>%

  mutate(

    lesion_height = case_when(

      str_starts(Lesion_level, "C")   ~ "cervical",

      str_starts(Lesion_level, "Th")  ~ "thoracic",

      str_starts(Lesion_level, "L")   ~ "lumbar",

      is.na(Lesion_level)             ~ NA_character_

    )

  )


VF_behav$lesion_height <- as.factor(VF_behav$lesion_height)


VF_behav_cervical_thoracic <-  VF_behav %>%

  filter(lesion_height %in% c("cervical", "thoracic"))


VF_behav_cervical_thoracic$lesion_height <- droplevels(VF_behav_cervical_thoracic$lesion_height)


table(VF_behav_cervical_thoracic$lesion_height)


# Demographic and clinical characteristics

table(VF_behav_cervical_thoracic$lesion_height)


#Test for difference of Sex between the two groups

table(VF_behav_cervical_thoracic$lesion_height, VF_behav_cervical_thoracic$Sex)

chisq.test(VF_behav_cervical_thoracic$Sex,VF_behav_cervical_thoracic$lesion_height,correct=FALSE)


#Age distribution

mean(VF_behav_cervical_thoracic$Age[VF_behav_cervical_thoracic$lesion_height=="cervical"])

sd(VF_behav_cervical_thoracic$Age[VF_behav_cervical_thoracic$lesion_height=="cervical"])


mean(VF_behav_cervical_thoracic$Age[VF_behav_cervical_thoracic$lesion_height=="thoracic"])

sd(VF_behav_cervical_thoracic$Age[VF_behav_cervical_thoracic$lesion_height=="thoracic"])


#Test for difference of age between the two groups

wilcox_test(Age~lesion_height,data=VF_behav_cervical_thoracic)


#TSI distribution

mean(VF_behav_cervical_thoracic$TSI[VF_behav_cervical_thoracic$lesion_height=="cervical"])

sd(VF_behav_cervical_thoracic$TSI[VF_behav_cervical_thoracic$lesion_height=="cervical"])


mean(VF_behav_cervical_thoracic$TSI[VF_behav_cervical_thoracic$lesion_height=="thoracic"])

sd(VF_behav_cervical_thoracic$TSI[VF_behav_cervical_thoracic$lesion_height=="thoracic"])


#Test for difference of TSI between the two groups

wilcox_test(TSI~lesion_height,data=VF_behav_cervical_thoracic)


#Compare pain level

mean(VF_behav_cervical_thoracic$pain[VF_behav_cervical_thoracic$lesion_height=="thoracic"])
```

```
sd(VF_behav_cervical_thoracic$pain[VF_behav_cervical_thoracic$lesion_height=="thoracic"])


mean(VF_behav_cervical_thoracic$pain[VF_behav_cervical_thoracic$lesion_height=="cervical"])

sd(VF_behav_cervical_thoracic$pain[VF_behav_cervical_thoracic$lesion_height=="cervical"])


t.test(pain~lesion_height,data=VF_behav_cervical_thoracic)


#Compare STAI trait levels

mean(VF_behav_cervical_thoracic$sum_stai_trait[VF_behav_cervical_thoracic$lesion_height=="thoracic"])

sd(VF_behav_cervical_thoracic$sum_stai_trait[VF_behav_cervical_thoracic$lesion_height=="thoracic"])


mean(VF_behav_cervical_thoracic$sum_stai_trait[VF_behav_cervical_thoracic$lesion_height=="cervical"])

sd(VF_behav_cervical_thoracic$sum_stai_trait[VF_behav_cervical_thoracic$lesion_height=="cervical"])


t.test(sum_stai_trait~lesion_height,data=VF_behav_cervical_thoracic)


#Compare STAI state levels

mean(VF_behav_cervical_thoracic$sum_stai_state[VF_behav_cervical_thoracic$lesion_height=="thoracic"])

sd(VF_behav_cervical_thoracic$sum_stai_state[VF_behav_cervical_thoracic$lesion_height=="thoracic"])


mean(VF_behav_cervical_thoracic$sum_stai_state[VF_behav_cervical_thoracic$lesion_height=="cervical"])

sd(VF_behav_cervical_thoracic$sum_stai_state[VF_behav_cervical_thoracic$lesion_height=="cervical"])


t.test(sum_stai_state~lesion_height,data=VF_behav_cervical_thoracic)


# Calculate Chi square test if there are significant differences in education level between the groups

aggregate(VF_behav_cervical_thoracic$Education,by=list(VF_behav_cervical_thoracic$lesion_height,VF_behav_cervical_thoracic$Ed
ucation),FUN=length)

contingency_table <- data.frame("thoracic"=c(1,5,2,2,3),"cervical"=c(0,4,2,2,4),row.names=c("Obligatorische_Schule","Berufsle
hre","Mittelschule","Hoehere_Berufsbildung","Universitaet"))

mosaicplot(contingency_table,color=TRUE)

chisq.test(contingency_table) #no difference


VF_behav_patient_ASIA <- VF_behav_patient_ASIA %>%

  left_join(VF_behav_cervical_thoracic %>% dplyr::select(ID, lesion_height),

            by = "ID")


#ISNCSCI scores

summary_stats_INSCSCI_cervical <- VF_behav_patient_ASIA %>%

  filter(lesion_height == "cervical") %>%

  summarise(

    mean_beruehrung = mean(Touch_total, na.rm = TRUE),

    sd_beruehrung = sd(Touch_total, na.rm = TRUE),

    mean_pinprick = mean(Pain_total, na.rm = TRUE),

    sd_pinprick= sd(Pain_total, na.rm = TRUE),

    mean_motorik = mean(Motor_total, na.rm = TRUE),

    sd_motorik = sd(Motor_total, na.rm = TRUE)

  )


summary_stats_INSCSCI_cervical
```

```
summary_stats_INSCSCI_thoracic <- VF_behav_patient_ASIA %>%
  filter(lesion_height == "thoracic") %>%
  summarise(
    mean_beruehrung = mean(Touch_total, na.rm = TRUE),
    sd_beruehrung = sd(Touch_total, na.rm = TRUE),
    mean_pinprick = mean(Pain_total, na.rm = TRUE),
    sd_pinprick= sd(Pain_total, na.rm = TRUE),
    mean_motorik = mean(Motor_total, na.rm = TRUE),
    sd_motorik = sd(Motor_total, na.rm = TRUE)
  )

summary_stats_INSCSCI_thoracic

wilcox.test(Touch_total~lesion_height,data=VF_behav_patient_ASIA)
wilcox.test(Pain_total~lesion_height,data=VF_behav_patient_ASIA)
wilcox.test(Motor_total~lesion_height,data=VF_behav_patient_ASIA)

#Compare sum of correct words
#Check assumptions for t-test
leveneTest(VF_behav_cervical_thoracic$Sum_Correctwords, VF_behav_cervical_thoracic$lesion_height)

#Normality assumption
by(VF_behav_cervical_thoracic$Sum_Correctwords, VF_behav_cervical_thoracic$lesion_height, shapiro.test) #normal

t.test(Sum_Correctwords~lesion_height,data=VF_behav_cervical_thoracic) #p=0.218

ggplot(VF_behav_cervical_thoracic, aes(x = lesion_height, y = Sum_Correctwords)) +
  geom_boxplot(outlier.shape=NA) +
  geom_signif(comparisons = list(c("cervical", "thoracic")),
              annotations = "p=0.219",  #adapt p-value
              y_position = 150, textsize = 5) +
  labs(x = "Lesion level", y = "Sum of correct words") +
  geom_jitter(data = VF_behav_cervical_thoracic, aes(x = lesion_height, y = Sum_Correctwords),
              width = 0.15, height =0, size = 2, color = "black", alpha = 0.6) +
  theme_minimal() +
  scale_x_discrete(labels = c("Non-SCI" = "Non-injured controls", "SCI" = "Spinal cord injury")) +
  theme(plot.title = element_text(hjust = 0.5),
        axis.title = element_text(size = 17),
        axis.text = element_text(size = 15))

#Compare normative data
leveneTest(VF_behav_cervical_thoracic$count_PR_smallerequal_10, VF_behav_cervical_thoracic$lesion_height)  #homogeneity of va
riances is fulfilled
by(VF_behav_cervical_thoracic$count_PR_smallerequal_10,VF_behav_cervical_thoracic$lesion_height, shapiro.test) #not fulfilled

wilcox.test(count_PR_smallerequal_10~lesion_height,data=VF_behav_cervical_thoracic)  #p=0.7128

ggplot(VF_behav_cervical_thoracic, aes(x = lesion_height, y = count_PR_smallerequal_10)) +
  geom_boxplot(outlier.shape=NA)+
  labs(x = "Lesion level", y = "Number of below-average\n performances out of 5 letters") +
  geom_jitter(data = VF_behav_cervical_thoracic, aes(x = lesion_height, y = count_PR_smallerequal_10),
```

```r
              width = 0.15, height =0, size = 2, color = "black", alpha = 0.6) +
  theme_minimal() +
  theme(plot.title = element_text(hjust = 0.5),
        axis.title = element_text(size = 17),
        axis.text = element_text(size = 15))+
  geom_signif(comparisons = list(c("cervical", "thoracic")),
              annotations = "p=0.714",  #adapt p-value
              y_position = 5, textsize = 5)



# Individuals with and without antimuscarinics ----------------------------
table(VF_behav_patientonly$Antimuscarinics)
table(VF_behav_patientonly$Antimuscarinics,VF_behav_patientonly$Sex)

t.test(Sum_Correctwords~Antimuscarinics,data=VF_behav_patientonly) #p=0.522

ggplot(VF_behav_patientonly, aes(x = Antimuscarinics, y = Sum_Correctwords)) +
  geom_boxplot(outlier.shape=NA) +
  geom_signif(comparisons = list(c("0", "1")),
              annotations = "p=0.522",  #adapt p-value
              y_position = 140, textsize = 5) +
  labs(x = "SCI groups", y = "Sum of correct words") +
  geom_jitter(data = VF_behav_patientonly, aes(x = Antimuscarinics, y = Sum_Correctwords),
              width = 0.15, height =0, size = 2, color = "black", alpha = 0.6) +
  theme_minimal() +
  scale_x_discrete(labels = c("0" = "Without\n antimuscarinics", "1" = "With\n antimuscarinics")) +
  theme(plot.title = element_text(hjust = 0.5),
        axis.title = element_text(size = 17),
        axis.text = element_text(size = 15))

#Compare normative data
leveneTest(VF_behav_patientonly$count_PR_smallerequal_10, VF_behav_patientonly$Antimuscarinics)  #homogeneity of variances is fulfilled
by(VF_behav_patientonly$count_PR_smallerequal_10,VF_behav_patientonly$Antimuscarinics, shapiro.test) #not fulfilled

wilcox.test(count_PR_smallerequal_10~Antimuscarinics,data=VF_behav_patientonly)  #p=0.768

ggplot(VF_behav_patientonly, aes(x = Antimuscarinics, y = count_PR_smallerequal_10)) +
  geom_boxplot(outlier.shape=NA)+
  labs(x = "SCI groups", y = "Number of below-average\n performances out of 5 letters") +
  geom_jitter(data = VF_behav_patientonly, aes(x = Antimuscarinics, y = count_PR_smallerequal_10),
              width = 0.15, height =0, size = 2, color = "black", alpha = 0.6) +
  theme_minimal()+
  theme(plot.title = element_text(hjust = 0.5),
        axis.title = element_text(size = 17),
        axis.text = element_text(size = 15))+
  geom_signif(comparisons = list(c("0", "1")),
              annotations = "p=0.768",  #adapt p-value
              y_position = 5, textsize = 5)  +
    scale_x_discrete(labels = c("0" = "Without\n antimuscarinics", "1" = "With\n antimuscarinics"))
```

```
# ASIA -------------------------------------------------------------------

#Calculate mean of motor, pinprick and light touch score

VF_behav_patient_ASIA <- VF_behav_patient_ASIA[VF_behav_patient_ASIA$ID!="SCI-Cog_32",]  #drop SCI-Cog_32 because of tendon t
ransfer


mean(VF_behav_patient_ASIA$Touch_total)

sd(VF_behav_patient_ASIA$Touch_total)

mean(VF_behav_patient_ASIA$Pain_total)

sd(VF_behav_patient_ASIA$Pain_total)

mean(VF_behav_patient_ASIA$Motor_total)

sd(VF_behav_patient_ASIA$Motor_total)


#Investigate differences between the left and right side Motor score

ASIA_motorscore_long <- pivot_longer(VF_behav_patient_ASIA,

                        cols = c(Motor_left, Motor_right),

                        names_to = "Side",

                        values_to = "Motor_Score")


ASIA_motorscore_long$Side <- as.factor(ASIA_motorscore_long$Side)


t.test(VF_behav_patient_ASIA$Motor_left,VF_behav_patient_ASIA$Motor_right,paired=TRUE) #significant p=0.009


mean(VF_behav_patient_ASIA$Motor_left) #lower than right

mean(VF_behav_patient_ASIA$Motor_right)


ggplot(ASIA_motorscore_long, aes(x = Side, y = Motor_Score)) +

  geom_boxplot(outlier.shape = NA) +

  labs(y = "Motor Score") +

  geom_point(aes(group = ID), size = 2, alpha = 0.6) +

  geom_line(aes(group = ID), color = "gray60", alpha = 0.7) +

  theme_minimal()+

  scale_x_discrete(labels = c("Motor_left" = "Left", "Motor_right" = "Right"))+

  theme(plot.title = element_text(hjust = 0.5),

        axis.title = element_text(size = 17),

        axis.text = element_text(size = 15))+

  geom_signif(comparisons = list(c("Motor_left", "Motor_right")),

              annotations = "p=0.009",  #adapt p-value

              y_position = 51, textsize = 5)



#Investigate differences between the left and right side pinprick

ASIA_pinprickscore_long <- pivot_longer(VF_behav_patient_ASIA,

                                     cols = c(Pain_left, Pain_right),

                                     names_to = "Side",

                                     values_to = "Pinprick_Score")


ASIA_pinprickscore_long$Side <- as.factor(ASIA_pinprickscore_long$Side)


t.test(VF_behav_patient_ASIA$Pain_left,VF_behav_patient_ASIA$Pain_right,paired=TRUE) #not significant p=0.303
```

```r
ggplot(ASIA_pinprickscore_long, aes(x = Side, y = Pinprick_Score)) +
  geom_boxplot(outlier.shape = NA) +
  labs(y = "Pin prick Score") +
  theme_minimal()+
  geom_point(aes(group = ID), size = 2, alpha = 0.6) +
  geom_line(aes(group = ID), color = "gray60", alpha = 0.7) +
  scale_x_discrete(labels = c("Pain_left" = "Left", "Pain_right" = "Right"))+
  theme(plot.title = element_text(hjust = 0.5),
        axis.title = element_text(size = 17),
        axis.text = element_text(size = 15))+
  geom_signif(comparisons = list(c("Pain_left", "Pain_right")),
              annotations = "p=0.303",  #adapt p-value
              y_position = 57, textsize = 5)



#Investigate differences between the left and right side Touch score
ASIA_Touchscore_long <- pivot_longer(VF_behav_patient_ASIA,
                                     cols = c(Touch_left, Touch_right),
                                     names_to = "Side",
                                     values_to = "Touch_Score")

ASIA_Touchscore_long$Side <- as.factor(ASIA_Touchscore_long$Side)

t.test(VF_behav_patient_ASIA$Touch_left,VF_behav_patient_ASIA$Touch_right,paired=TRUE) #significant p=0.547

ggplot(ASIA_Touchscore_long, aes(x = Side, y = Touch_Score)) +
  geom_boxplot(outlier.shape = NA) +
  labs(y = "Light touch Score") +
  geom_point(aes(group = ID), size = 2, alpha = 0.6) +
  geom_line(aes(group = ID), color = "gray60", alpha = 0.7) +
  theme_minimal() +
  scale_x_discrete(labels = c("Touch_left" = "Left", "Touch_right" = "Right"))+
  theme(plot.title = element_text(hjust = 0.5),
        axis.title = element_text(size = 17),
        axis.text = element_text(size = 15))+
  geom_signif(comparisons = list(c("Touch_left", "Touch_right")),
              annotations = "p=0.547",  #adapt p-value
              y_position = 57, textsize = 5)



# Voxel-based morphomentry analysis ------------------------------------------------------------
VBM <- read_excel("D:/Main_study/VBM_Volume_measures.xlsx")
VBM$Group <- as.factor(VBM$Group)
VBM$Sex <- as.factor(VBM$Sex)

#test for volume differences in regions which showed significant differences in the fMRI data
model <- lm(Left_insula_tot ~ Group + TIV + Age, data=VBM)
summary(model)

model <- lm(`Left SFG superior frontal gyrus` ~ Group + TIV + Age, data=VBM)
```

```r
summary(model)


model <- lm(`Right Putamen` ~ Group + TIV + Age, data=VBM)

summary(model)



ggplot(VBM, aes(x = Group, y = Left_insula_tot)) +

  geom_boxplot(outlier.shape = NA) +

  labs(y = "Volume left insula [ml]") +

  geom_point(aes(group = ID), size = 2, alpha = 0.6) +

  geom_line(aes(group = ID), color = "gray60", alpha = 0.7) +

  theme_minimal() +

  theme(plot.title = element_text(hjust = 0.5),

        axis.title = element_text(size = 17),

        axis.text = element_text(size = 15))+

  geom_signif(comparisons = list(c("SCI", "Non-SCI")),

              annotations = "p=0.769",  #adapt p-value

              y_position = 7.3, textsize = 5)


ggplot(VBM, aes(x = Group, y = `Right Putamen`)) +

  geom_boxplot(outlier.shape = NA) +

  labs(y = "Volume right putamen [ml]") +

  geom_point(aes(group = ID), size = 2, alpha = 0.6) +

  geom_line(aes(group = ID), color = "gray60", alpha = 0.7) +

  theme_minimal() +

  theme(plot.title = element_text(hjust = 0.5),

        axis.title = element_text(size = 17),

        axis.text = element_text(size = 15))+

  geom_signif(comparisons = list(c("SCI", "Non-SCI")),

              annotations = "p=0.119",  #adapt p-value

              y_position = 5.5, textsize = 5)


ggplot(VBM, aes(x = Group, y = `Left SFG superior frontal gyrus`)) +

  geom_boxplot(outlier.shape = NA) +

  labs(y = "Volume left superior frontal gyrus [ml]") +

  geom_point(aes(group = ID), size = 2, alpha = 0.6) +

  geom_line(aes(group = ID), color = "gray60", alpha = 0.7) +

  theme_minimal() +

  theme(plot.title = element_text(hjust = 0.5),

        axis.title = element_text(size = 17),

        axis.text = element_text(size = 15))+

  geom_signif(comparisons = list(c("SCI", "Non-SCI")),

              annotations = "p=0.786",  #adapt p-value

              y_position = 17, textsize = 5)
```

References

1. Faul F, Erdfelder E, Buchner A, Lang AG. Statistical power analyses using G*Power 3.1: Tests for correlation and regression analyses. *Behav Res Methods*. 2009;41(4):1149-1160. doi:10.3758/BRM.41.4.1149

2. Korkmaz N, Ma G, mc G, rkmen A, lu zlem, lmaz B. Cognitive functions in individuals with spinal cord injury: Do symptoms of autonomic dysreflexia or orthostatic hypotension have an effect? *Ann Med Res*. 2022;29(10):1. doi:10.5455/annalsmedres.2022.06.198

3. Aschenbrenner S, Tucha O, Lange KW. *RWT Regensburger Wortflüssigkeits-Test*. Hogrefe; 2000.

4. Koo TK, Li MY. A Guideline of Selecting and Reporting Intraclass Correlation Coefficients for Reliability Research. *J Chiropr Med*. 2016;15(2):155-163. doi:10.1016/j.jcm.2016.02.012

5. Wagner S, Sebastian A, Lieb K, Tüscher O, Tadić A. A coordinate-based ALE functional MRI meta-analysis of brain activation during verbal fluency tasks in healthy control subjects. *BMC Neurosci*. 2014;15(1):19. doi:10.1186/1471-2202-15-19

6. Ruxton K, Woodman RJ, Mangoni AA. Drugs with anticholinergic effects and cognitive impairment, falls and all-cause mortality in older adults: A systematic review and meta-analysis. *Br J Clin Pharmacol*. 2015;80(2):209-220. doi:10.1111/bcp.12617

7. Mefford J, Witte JS. The Covariate's Dilemma. *PLoS Genet*. 2012;8(11):e1003096. doi:10.1371/journal.pgen.1003096
